# USENSE: A proof‐of‐concept self‐screening tool for home‐based recurrent urinary tract infection management

**DOI:** 10.1002/btm2.70038

**Published:** 2025-07-10

**Authors:** Antra Ganguly, Ujjaini Basu, Varun Gunda, Ashwin Krishnan, Pranav Ramesh, Kush Jivnani, Arjun Raghuram, Shashank Bhagavatula, Sifa Khan, Philippe Zimmern, Nicole De Nisco, Shalini Prasad

**Affiliations:** ^1^ Department of Bioengineering University of Texas at Dallas Richardson Texas USA; ^2^ Department of Biological Sciences University of Texas at Dallas Richardson Texas USA; ^3^ Department of Healthcare Management University of Texas at Dallas Richardson Texas USA; ^4^ Department of Neuroscience University of Texas at Dallas Richardson Texas USA; ^5^ Department of Urology University of Texas Southwestern Medical Center Dallas Texas USA

**Keywords:** interleukin 8 (IL8), lipopolysaccharide (LPS), machine learning, prostaglandin E2 (PGE2), recurrent urinary tract infection (rUTI), UTI prognosis

## Abstract

In this work, we report a novel proof‐of‐concept biosensing diagnostic tool for the multiplexed electrochemical quantitation of a unique combination of three UTI‐relevant biomarkers, Prostaglandin E2 (PGE2), Interleukin‐8 (IL‐8), and Lipopolysaccharide (LPS), in unfiltered human urine. The proposed device, called USENSE, integrates lateral flow microfluidic channels, a gold‐based sensor array for quantifying PGE2, IL‐8, and LPS levels, and a random forest machine learning model for reliable diagnosis of UTI. The device is unique as it not only acts as a diagnostic device but also provides information on UTI by providing a risk score for UTI recurrence. USENSE is culture‐free and label‐free, requires no sample preparation at the user end, and can be adapted for use in home‐based self‐screening. In less than 5 minutes, USENSE directly measures the urinary concentration of PGE2, IL‐8, and LPS and provides a UTI severity state classification: 0 = Healthy, 1 = Asymptomatic Bacteriuria, 2 = Symptomatic; low risk of relapse, 3 = Symptomatic; high risk of UTI relapse. In postmenopausal women, the PGE2, IL8, and LPS concentrations measured via the device correlated well with the levels measured using traditional enzyme‐linked immunosorbent assay (ELISA). Our machine learning diagnostic model allowed for UTI diagnosis with 93% test accuracy and UTI prognosis state classification with >84% accuracy for the human urine samples tested. Further development of USENSE for clinical and home‐based use could create a paradigm shift in point‐of‐care UTI diagnostics by allowing timely intervention and minimizing unwarranted empirical administration of antibiotics.

**Translational Impact Statement**

USENSE is a novel biosensing tool for the diagnosis and prognosis of recurrent urinary tract infection (rUTI), and the prediction of the risk of rUTI recurrence. It is culture‐free, requires no sample preparation, and is especially invaluable for patients who do not present like classic index patients. It can also differentiate UTI from ASB. Its easy‐to‐use user interface makes it suitable for self‐triage of UTI severity and routine at‐home monitoring. USENSE can enable improved antibiotic stewardship, reduction in costs, and increased treatment success.

## 1 INTRODUCTION

**FIGURE 1:**
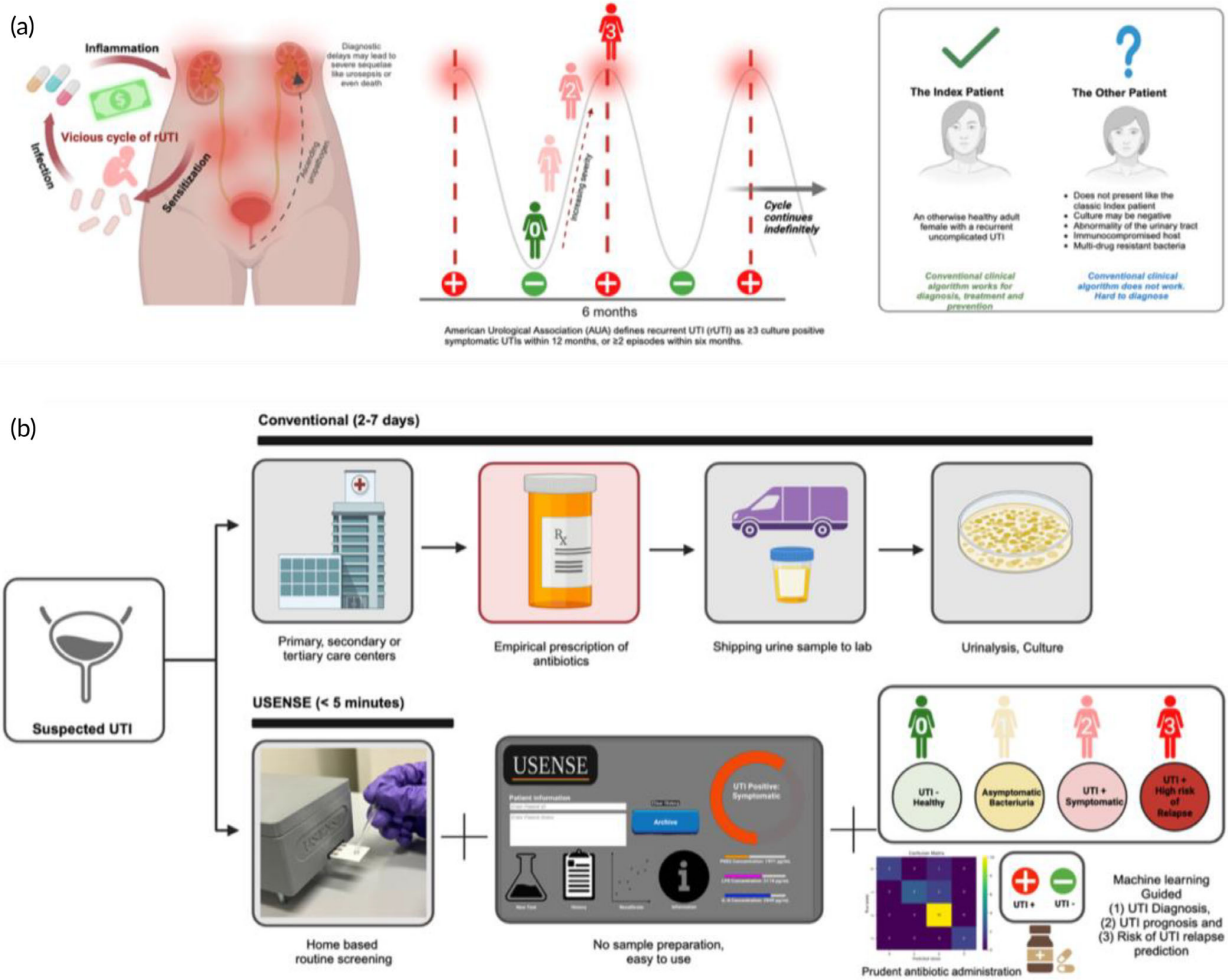
(a) The disease burden of rUTI adversely impacts patient quality of life and results in a significant financial burden for patients and their families. (b) USENSE pathways versus the current clinical pathway. The USENSE platform can diagnose UTIs and enable UTI prognosis through state and severity classification.

Hooton et al. define urinary tract infection (UTI) as when a significant concentration of bacteriuria is present in a patient with symptoms attributable to the urinary tract and no other source.^1^ Thus, the diagnosis of UTI depends not only on the presence of the causative pathogen, which is most commonly *Escherichia coli*, but also on the presence of symptoms (Figure 1(a)). Accordingly, the gold standard for UTI diagnosis relies on accurate symptom reporting and clinical urine culture for pathogen identification.^2^ However, in addition to a 24–48‐h diagnostic window, urine culture is prone to false negative results as it only detects limited species of potential uropathogens. The commonly used diagnostic threshold for UTI diagnosis is 10^4^ colony‐forming units per milliliter (CFU/mL); however, 30–50% of women with symptomatic UTIs are found to have bacterial counts as low as 10^2^ CFU/mL.^3^ On the other hand, a high bacterial urine count, termed bacteriuria, also does not imply a UTI episode. Some patients with bacteriuria do not present with symptoms. This condition is termed asymptomatic bacteriuria (ASB), for which antibiotic treatment should be avoided as it may lead to adverse events like the acquisition of drug‐resistant pathogens and *Clostridium difficile* infection.^4^ Thus, for reliable diagnosis, improved antibiotic stewardship, and reduced patient and provider frustration, it is crucial for new diagnostic paradigms to function at the point‐of‐care (POC) and account for both the infection and the resulting inflammatory response, especially for patients who do not present like classic index patients (Figure 1(a)).

UTIs are often associated with frequent recurrence, especially in postmenopausal women.^5^ The American Urological Association (AUA) defines recurrent UTI (rUTI) as ≥ three culture‐positive symptomatic UTI episodes within 12 months or ≥two episodes within six months.^6^ Nearly 60% of women will suffer from UTI at least once in their lifetime, and approximately 20%–40% of women who have had one previous episode are likely to experience an additional episode, 25%–50% of whom will experience multiple recurrent episodes.^6^ Excessive inflammation and injury in the bladder due to infection may result in bladder sensitization, making individuals prone to severe rUTI even after antibiotic therapy.^7^ Thus, patients are often trapped in a vicious cycle of rUTI, which may last for many years and adversely affect quality of life, mental health (e.g., low self‐esteem, depression), and significantly reduce physical and social functions (Figure 1(a)).^8^ UTI prognosis and the prediction of rUTI risk are, therefore, critical for clinicians to develop informed UTI management strategies and to improve antibiotic stewardship.

In this work, we discuss the proof of concept and development of USENSE, a novel biosensing tool for recurrent urinary tract infection (rUTI) that not only acts as a diagnostic device but also provides a prognosis by predicting the risk of rUTI recurrence. USENSE relies on the multiplexed electrochemical quantitation of a unique combination of three UTI‐relevant biomarkers, Prostaglandin E2 (PGE2), Interleukin‐8 (IL‐8), and Lipopolysaccharide (LPS), in unfiltered human urine. It is culture‐free and label‐free, requires no sample preparation at the user end, and can be adapted for use in home‐based self‐screening. Besides providing UTI diagnosis, it also provides a prediction for the risk of relapse and is capable of discriminating between UTI and ASB. USENSE has a simple and easy‐to‐use user interface for routine screening and self‐triage of UTI severity by rUTI patients for at‐home monitoring. This will enable timely and appropriate prescription of antibiotics, reduction in costs, and increased treatment success.

For the development of the proof‐of‐concept USENSE device for improved POC UTI diagnosis and prognosis, we chose a bacterial biomarker, i.e., *E. coli* Lipopolysaccharide (LPS), and two inflammatory biomarkers specific and sensitive to UTI, viz., Prostaglandin E2 (PGE2) and Interleukin 8(IL‐8). PGE2 is a product of cyclooxygenase‐2 (COX‐2), and studies in mice have shown that COX‐2‐mediated inflammation sensitizes the bladder to recurrent UTI (rUTI).^9^ Ebrahimzadeh et al. have shown activated COX‐2 expression in bladder urothelium and elevated urinary PGE2 levels in postmenopausal women with rUTI. Urinary PGE2 was also shown to be predictive of recurrent UTI, indicating that it can also serve as a prognostic biomarker.^10^ MIP‐2, a functional orthologue of human IL‐8 that is involved in neutrophil trafficking, is the first detected cytokine in mouse models of UTI.^11^ Multiple studies in human cohorts have observed elevated urinary IL‐8 levels in UTI cases versus controls.^12^, ^13^, ^14^ Importantly, a recent study demonstrated that a diagnostic model including urinary IL‐8 and PGE2 was able to differentiate cases from controls with high specificity and sensitivity.^12^ LPS is a macromolecular glycolipid^15^, ^16^ and the central component of the outer leaflet of the outer membrane of Gram‐negative bacteria like *E. coli, Klebsiella pneumoniae*, *a*nd *Proteus mirabilis*, which together cause >85% of UTIs.^17^ LPS levels in the urine can be correlated to the bacterial load in a patient and, therefore, can serve as a marker of Gram‐negative bacteriuria.^15^ Hence, for multiplexed analysis, our USENSE biomarker panel comprised inflammatory markers IL8^18^ and PGE2 along with LPS as a Gram‐negative bacterial biomarker (Ebrahimzadeh et al., 2021b). While a single biomarker is insufficient for UTI diagnosis, we hypothesize that a combination of these three biomarkers will allow accurate UTI diagnosis and will distinguish ASB from UTI in clinical settings. Though PGE2,^19^, ^20^, ^21^, ^22^, ^23^ IL‐8,^24^, ^25^, ^26^, ^27^, ^28^ and LPS^15^, ^29^, ^30^, ^31^, ^32^ have been used to develop other biosensors for body fluid analysis for various disease models, this work is the first to demonstrate their combinatorial use for multiplexed quantification through label‐free electrochemical biosensing in human urine (with no sample preparation at the user end) for UTI and rUTI diagnosis and management and risk of relapse prediction.

USENSE is one‐of‐a‐kind and invaluable for reliable diagnosis and improved antibiotic stewardship, and is crucial for new diagnostic paradigms to function at the point‐of‐care (POC), especially for patients who do not present like classic index patients, as discussed before. The developed biosensor shows several advantages over the current UTI detection methods including (i) rapid response time, (ii) multiplexed quantitative output, (iii) highly specific and sensitive non‐culture‐based affinity biosensing, (iv) portability and low cost due to compatibility with microfabrication and miniaturized electronics, (v) independent of the causative pathogen, (vi) little to no sample preparation, and (vii) label‐free, easy to use operation. Instead of being phenotype‐driven, our diagnostic method is endotype‐driven and takes into consideration the dynamic biological variability due to genetic predisposition, treatment response, and non‐linearity of triggered biological pathways, which can be potentially converted to direct, easy‐to‐interpret digital outcomes (suitable for home‐based environments).

UTIs are expensive to manage and are associated with a severe financial burden, costing about $2 billion annually in the United States alone.^2^ We developed a new, cost‐effective solution and disposable design for our USENSE sensor to alleviate this financial stress. It is envisioned that patients will routinely use the disposable USENSE sensor to monitor and manage symptoms and upcoming episodes of infection (Figure 1(b)). This will empower them to schedule timely visits to the clinic or hospital and enable clinicians to administer antibiotics appropriately and avoid empiric prescriptions due to delayed culture results. This is especially important for resource‐challenged settings, where therapeutic options may be limited, and clinical lab results may be delayed. Through the powerful combination of critical UTI‐relevant biomarkers and non‐faradaic, label‐free, electrochemical measurement coupled with machine learning analytics, USENSE can output UTI diagnosis and prognosis for a given patient sample within a five‐minute diagnostic window. To our knowledge, this work is the first of its kind, which, besides providing UTI diagnosis, also provides a prediction for the risk of relapse and is capable of discriminating between UTI and ASB. The developed simple and easy‐to‐use user interface further makes USENSE suitable for routine screening and self‐triage of UTI severity by rUTI patients in the comfort of their homes.

## 2 EXPERIMENTAL SECTION

### 2.1 Materials and reagents

Uric acid, calcium chloride, sodium citrate tribasic dihydrate, creatinine, ammonium chloride, potassium oxalate monohydrate, magnesium sulfate heptahydrate, sodium phosphate monobasic dihydrate, sodium phosphate dibasic dihydrate, paraffin wax, and HPLC‐purified PGE2 antigen were purchased from Sigma‐Aldrich (St. Louis, MO). Sodium sulfate, urea, potassium chloride, and sodium chloride were obtained from Fisher Scientific (Waltham, MA). Monoclonal α‐PGE2 antibody was obtained from Arbor Assays (Ann Arbor, MI). Monoclonal IL‐8 antibody and antigen were purchased from Abcam (Waltham, MA). Polyclonal LPS antibody and antigen, the crosslinker DSP (dithiobis (succinimidyl propionate)), DMSO (dimethyl sulfoxide), and PBS (phosphate buffer saline) were obtained from Thermo Fisher Scientific (Waltham, MA). Pooled human urine samples (pH ~6.5) were purchased from Lee Biosolutions (St. Louis, MO). Artificial urine was prepared using the recipe described by Sarigul et al.^21^, ^23^, ^33^ Silhouette Cameo 4 cutter printer and Silhouette Studio software were obtained from Silhouette America (Lindon, UT). AutoCAD software was obtained from Autodesk Inc. (San Francisco, CA). The Whatman Fusion 5 membrane (lateral flow membrane) was purchased from Cytiva (Global Life Sciences Solutions USA LLC, Marlborough, MA). Plain paper copier transparency film sheets were purchased from Apollo (Lake Zurich, IL). Kapton tape was purchased from DuPont (Wilmington, DE). ArCare 7815 adhesive backing was purchased from Adhesives Research (Glen Rock, PA). Pro‐Wax100 wax heater was purchased from PRO Car Beauty Products (Tustin, CA). The heat press was purchased from Geo Knight (Brockton, MA).

### 2.2 Working electrode functionalization and Ab conjugation

A sensor design was created in AutoCAD with a standard three‐electrode system (working, counter, and reference) for each of the three channels. 5 μL of 10 mM DSP was dispensed onto each of the gold working electrodes. The sensor cartridges were subsequently placed in a container, wrapped in aluminum foil, and followed by a 1.5‐h incubation in the dark. 5 μL of PBS was added prior to dispensing LPS, IL‐8, and PGE2 Abs onto the working electrodes with one Ab per channel. Different combinations of Ab locations (left, central, and right channels) were tested. This was followed by a 1‐h incubation at 4°C. Electrodes were treated with 5 μL of SuperBlock™ Blocking buffer (Thermo Fisher Scientific (Waltham, MA)) and left for 15 min. Sensor cartridges were stored at 4°C until use.

### 2.3 Experimental design of human subject studies and ELISA studies

Samples were selected from archived midstream clean catch urine samples that were obtained from patients at the University of Texas Southwestern Medical Center Urology clinic. Urine was immediately chilled and aseptically processed following storage in liquid nitrogen within 2 h. Selected patients passed the exclusion criteria of PVR > 150 mL, >stage 2 bladder prolapse, immune suppression, history of catheterization, and surgery less than a month prior to sample collection. The clinical samples were collected as part of approved Institutional Review Board protocols (STU 082010–016, MR 17–120). Quantification of PGE2, IL‐8, and LPS was done by ELISA. PGE2 levels were measured by a highly sensitive kit from Enzo. Optical density (O.D.) was read at 405 nm. IL‐8 levels were measured using the Human IL‐8/CXCL8 ELISA kit (Sigma‐Aldrich) with O.D. measured at 450 nm. LPS concentration was determined by a kit from Abclonal with O.D. measured at 450 nm. All O.D. measurements were done with the Synergy H1 plate reader (BioTek).

### 2.4 Electronic system design and integration

A Windows application was developed to run simultaneous EIS tests to determine LPS, PGE2, and IL‐8 levels in urine. It is compatible with personal devices with a connection to a PalmSens EmStat MUX 16 multiplexer. The GUI application was developed using the Unity game engine and PalmSens C# SDK for.NET to begin and interpret the EIS reading and create an environment where pertinent information can be stored. The ease of use of the application provides an avenue through which UTI testing can be done fast and reliably in the clinical setting. The GUI development has been discussed in further detail in the Data S1.

### 2.5 Machine learning model development

To be truly useful as a self‐triage and self‐monitoring home‐based POC tool, the results from the USENSE platform needed to be easy to interpret and actionable. To do this, we designed a machine learning algorithm by building on our previous work of DiGEST^23^ platform. Similar to our previous work, the machine learning algorithms were coded in Python in Google's CoLab platform (quad‐core Intel Xeon processor, Tesla V100‐SXM2‐16GB GPU). The random forest models for the diagnostic and prognostic cases were built using the Sklearn library (bootstrapping was done randomly with replacement). The feature importance score available in the Sklearn random forest model was used to rank the features based on their importance to create the reduced random forest models. Data visualization was done using Matplotlib and Seaborn functions.

## 3 RESULTS AND DISCUSSION

### 3.1 Design of the multiplexed lateral flow electrochemical biosensor cartridge

**FIGURE 2:**
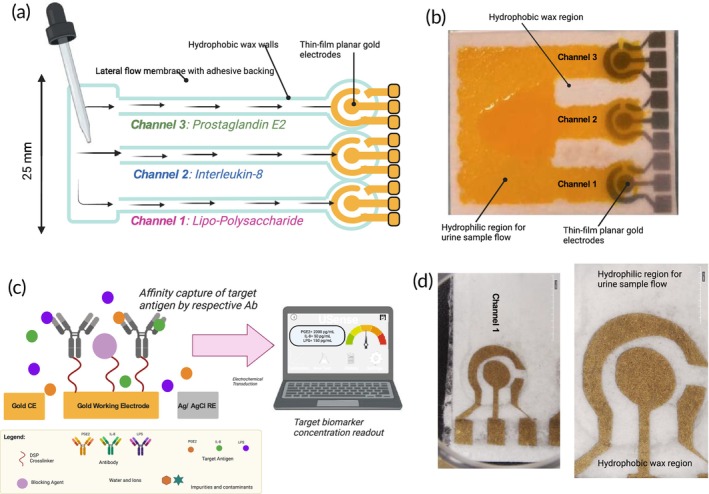
(a) Schematic showing the design of the multiplexed USENSE disposable cartridge, (b) Dye test result indicating the three channels and the hydrophobic wax regions. 0.2 mL of sample completely covers the electrodes. There is no inter‐bleeding between the three channels. (c) Schematic showing the affinity electrochemical biosensing principle for IL8, PGE2, and LPS quantification; (d) Sensor micrographs showing the gold electrodes, hydrophobic walls, and hydrophilic channels.

Key components of the USENSE platform are (1) a disposable, multiplexed microfluidic gold biosensor cartridge fabricated on a Cytiva substrate^34^, ^35^ via gold electron beam deposition and (2) a 3D printed electronic reader that incorporates a portable multichannel potentiostat for sensor data acquisition via USB communication (see Data S1). The sensor array consists of three planar, three‐electrode gold sensors (Figure 2). The working electrode is immobilized with the specific antibody corresponding to the target antigen of interest. The design, optimization, and quality control of the sensor fabrication process has been discussed in detail in the Data S1. Considering that the potential end users of this technology include patients who are not medical professionals and will use the device at their homes, we have designed a simple, interactive, and easy‐to‐use Graphical User Interface (GUI) for USENSE that is discussed in detail in the Data S1. The disposable biosensor cartridge fabrication protocol has been designed to be cost‐effective and easily reproducible (Figures S1–S3). To realize rapid, simultaneous, and label‐free IL8, PGE2, and LPS detection in situ, the microfluidic module comprises a sample loading reservoir where the test urine sample (0.2 mL) is dropped. The loaded sample rapidly flows down three channels to reach the antibodies immobilized on the individual working electrodes, separated by hydrophobic walls (Figure 2). The hydrophobic walls and channels were created using paraffin wax to keep the design cost‐effective. The optimization process has been discussed in detail in the methods section.

### 3.2 Choice of biomarkers for UTI diagnosis and prognosis

For our proof‐of‐concept sensor design, we chose to develop a triplex sensor capable of quantifying urinary Prostaglandin E2 (PGE2), Interleukin 8 (IL‐8), and Lipopolysaccharide (LPS) that would allow discrimination between healthy controls, individuals with UTI, and individuals with asymptomatic bacteriuria. These biomarkers, two inflammatory (PGE2, IL‐8) and one bacterial (LPS), have shown promise in UTI diagnosis, UTI prognosis, or in the detection of bacteriuria. PGE2, which is the product of the COX‐2 enzyme, has been shown to be a promising diagnostic and prognostic marker that can predict the risk of relapse of UTI^10^ (Figure S4). IL‐8 is a pro‐inflammatory cytokine that has been shown to be elevated in patients with active UTI but not with asymptomatic bacteriuria^13^, ^14^ (Figure S4). Furthermore, a recent study by Ebrahimzadeh et al. found that a diagnostic model based on urinary PGE2 and IL‐8 concentration was able to distinguish cases from controls with high accuracy and a low misclassification rate.^12^ We reasoned that LPS, which comprises the outer leaflet of the outer membrane of Gram‐negative bacteria, would be a direct marker for Gram‐negative uropathogens like *Escherichia coli* and *Klebsiella pneumoniae* that cause the majority of UTIs and are commonly found in women with ASB.^36^, ^37^ We reasoned that including a bacterial biomarker like LPS would be essential to identify cases of ASB where bacteria are present in the urine without an associated immune response (i.e., elevated PGE2 and IL‐8). A truth table was used as a guiding logic to determine the Boolean equation for the USENSE sensor, and the disease classification states for diagnosis and prognosis were defined as shown in Figure S5.

The physiologically relevant ranges for the biomarkers were determined by ELISA quantification of the samples of a cohort of post‐menopausal women. The ranges were found to be 500–4000 pg/mL for PGE2, 1–2000 pg/mL for IL‐8, and 1–320 pg/mL for LPS. The diagnostic cutoffs were measured to be 158 pg/mL for IL‐8, 1800 pg/mL for PGE2, and 40 pg/mL for LPS. Kruskal–Wallis analyses in Figure S6 show that no single biomarker is capable of distinguishing between all 4 states. For example, IL8 is capable of distinguishing between states 0 and 2 and states 0 and 3, but not states 1 and 3 or states 0 and 1. PGE2 is capable of distinguishing between 0 and 3. LPS is capable of distinguishing between 0 and 1, 0 and 2, and also 0 and 3. In this way, it can be concluded that in order to truly achieve state classification for UTI prognosis, a single biomarker measurement is insufficient, and we need to use this combination of biomarkers to be more effective.

### 3.3 Characterization and validation of binding chemistry

**FIGURE 3:**
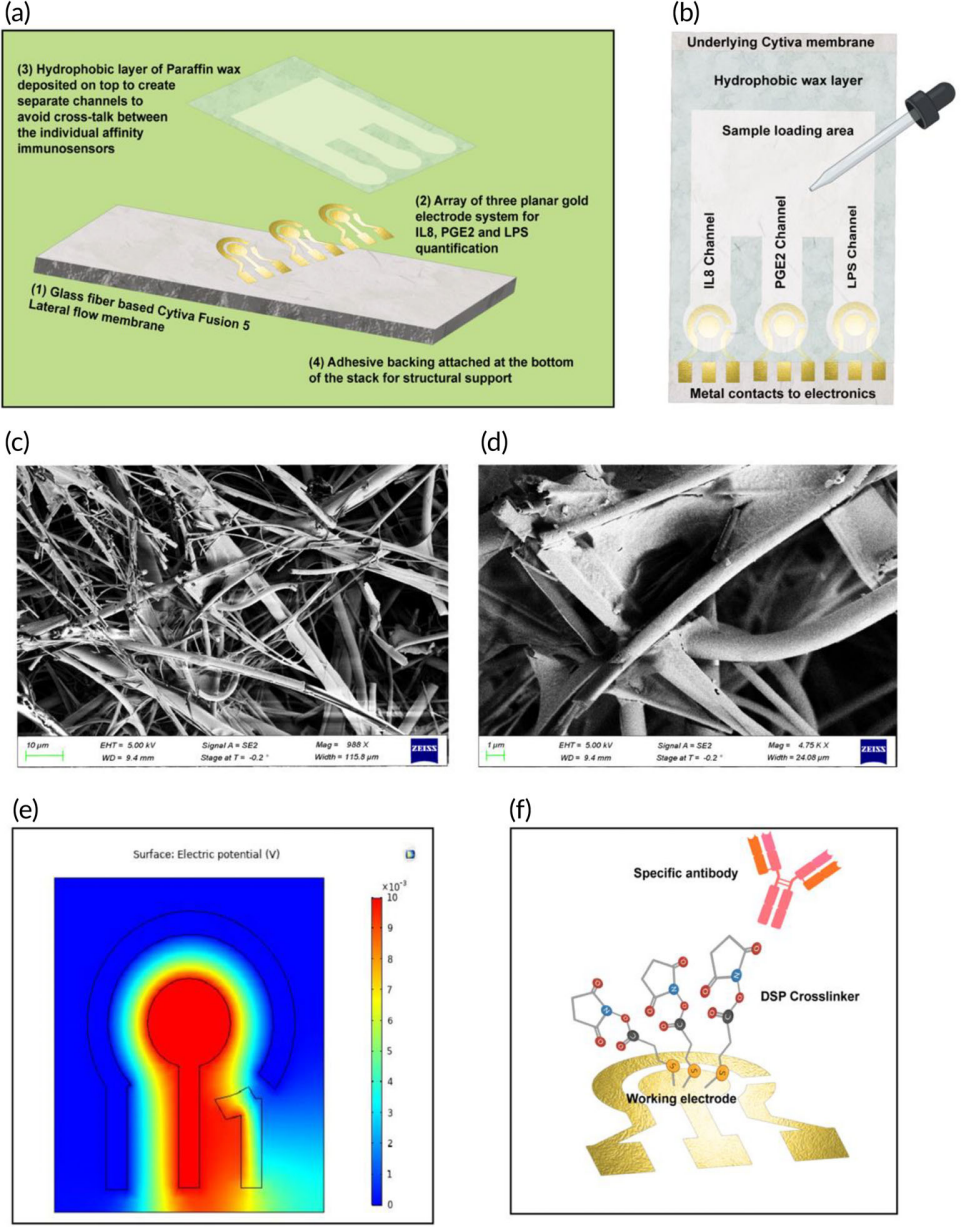
(a) USENSE sensor cartridge stack, (b) schematic highlighting the sensor cartridge components, (c) and (d) are SEM images of the gold deposited on the porous Cytiva lateral flow membrane, allowing for high surface roughness, high surface area, and surface reactivity for binding. (e) shows the COMSOL‐based finite element analysis of the electric potential for an individual 3‐electrode sensor. This shows the distribution of the applied electric field across the WE, RE, and CE. WE acts as the source and CE acts as the sink for the electric current flow. (f) highlights the biosensor assay components for an individual immunosensor.

The USENSE cartridge has been designed as three immunosensors that perform simultaneous quantification of three target analytes at a time. The USENSE sensor cartridge stack has been shown in Figure 3(a) and the sensor cartridge components have been highlighted in Figure 3(b). The biosensing is achieved by the affinity capture principle in which the target antigens (PGE2, IL‐8, and LPS) expressed in the urine sample preferentially bind to their corresponding highly specific antibodies (monoclonal Ab for PGE2, IL‐8, and polyclonal Ab for LPS). These antibodies are attached to the gold surface by strong thiol bonds using a homobiofunctional and amine‐reactive thiol crosslinker, DSP (dithiobis(succinimidyl propionate)).^21^ Each of the three immunosensors in the corresponding three channels is in the form of a standard planar three‐electrode system comprising a working electrode, a reference electrode, and a counter electrode. The DSP self‐assembled monolayer formation and the antibody–antigen binding chemistry occur at the working electrode. Before proceeding with the biosensing experiments, the three‐electrode sensor design was optimized using finite element analysis using COMSOL Multiphysics software (Figure 3(e)).^28^ The porosity of the Cytiva Fusion 5 membrane allows for a high surface area to volume ratio, allowing high sensitivity EIS biosensing.^21^, ^28^, ^38^, ^39^, ^40^, ^41^, ^42^ The porous nature of the membrane has been depicted in Figure 3(c),(d). Further characterization of the binding chemistry was done using Attenuated Total Reflectance Fourier Transform Infrared Spectroscopy (Figures S7–S9) and UV–Vis analysis (Figure S10) for each biomarker assay (discussed in detail in the Data S1). The results of the surface charge behavior analysis of the elements of the assay stack have also been discussed in the Data S1 (Figure S11).

### 3.4 Electrochemical performance testing of USENSE biosensor for Interleukin 8, Prostaglandin E2, and Lipopolysaccharide quantification in urine

We studied the interfacial modulation of the electrical properties, in the form of impedance change, due to Ab‐Ag binding using the powerful AC‐based electrochemical technique of Electrochemical Impedance Spectroscopy. Since no redox mediators or labeling was done, the three electrochemical immunosensors on each cartridge operate in the non‐faradaic mode, where the modulation of the interfacial capacitance as a function of binding was used for signal transduction. Single‐frequency EIS measurements (100 Hz) recorded impedance measurements taken using a potentiostat (Gamry Instruments, Warminster, PA) after applying an AC excitation signal of 10 mV rms. Voltage for the EIS protocol was optimized to yield an ohmic response without compromising biomolecular stability.^21^, ^22^, ^23^ The maximum capacitive response, indicated by the highest negative phase, was attained at nearly 100 Hz in our previous work.^21^, ^23^ Thus, 100 Hz was chosen to study the modulation of impedance for analysis. This protocol was followed for the detection of PGE2, IL‐8, and LPS molecules in spiked pooled human urine. We achieved a dynamic range of 10–6250 pg/mL for IL8, 250–5000 pg/mL for PGE2, and 1–320 pg/mL for LPS. As mentioned before, the diagnostic threshold was defined as 158 pg/mL for IL‐8, 1800 pg/mL for PGE2, and 40 pg/mL for LPS. Figure S12 shows the dose–response curve for PGE2, IL‐8, and LPS detection in spiked pooled human urine. As expected, the modulus of impedance decreases in a dose‐dependent manner as the system becomes increasingly capacitive due to Ab‐Ag binding, due to the variation in the dielectric constant at the gold electrode–urine buffer interface. Curve fitting using 4 Parameter Logistic Regression analysis (dotted line) gave an *R* ^2^ > 0.95 for all the biomarkers as shown in Figure S12. The impedance change at 100 Hz relative to the baseline/blank dose was studied for *n* = 3 inter‐sensor and *n* = 3 intra‐sensor replicates (see Data S1). In this way, the USENSE multiplexed biosensor performance was validated for simultaneous urine PGE2, IL8, and LPS detection.

### 3.5 Precision

We also studied the precision of the USENSE system by evaluating the precision of the individual electrochemical immunosensors. We did this by evaluating the coefficient of variation (CV%), which was calculated by averaging over all the replicates (*n* = 3 inter‐sensor and *n* = 3 intra‐sensor replicates) as a function of the IL‐8, PGE2, and LPS levels spiked in urine samples (Figure S13). The CV% for all the biomarkers was found to be under 20%, which is accepted by the Clinical Laboratory Standards Institute (CLSI) guidelines,^43^, ^44^, ^45^ indicating the USENSE is capable of precise biomarker‐level quantification.

### 3.6 Human subject testing

**FIGURE 4:**
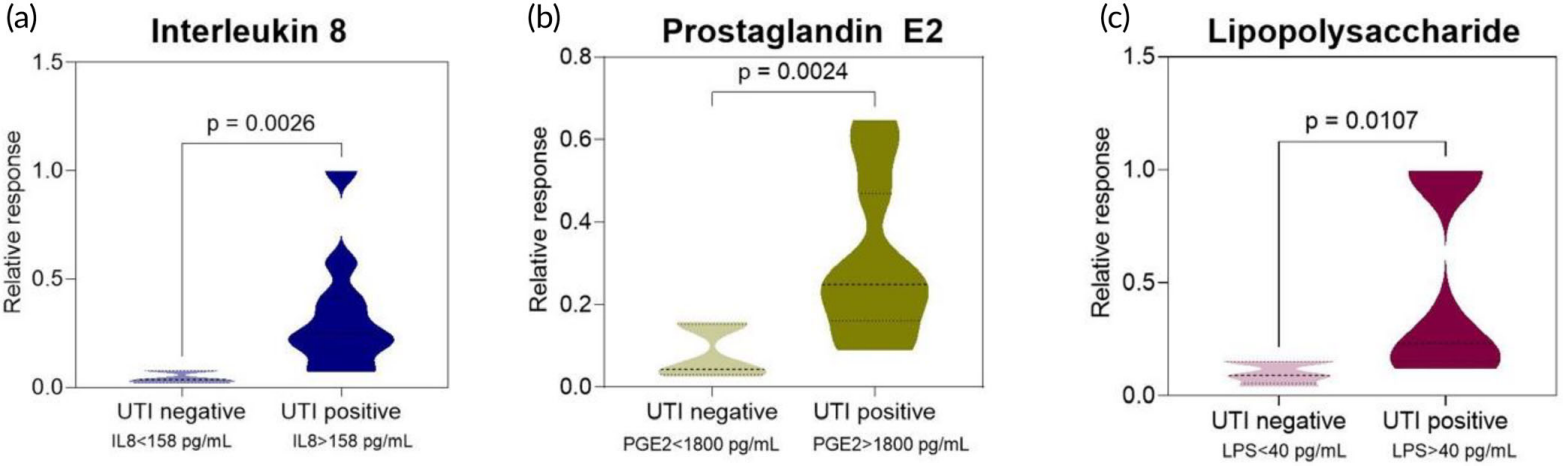
*T*‐test analysis (two‐tailed, unpaired, *α* = 0.05) for sensor response for UTI negative and UTI positive patient samples for (a) IL8, (b)PGE2, and (c) LPS using thresholds mentioned before.

The proposed application and scope of the developed sensor for human subject testing are depicted in Figure 1(b). After evaluating the sensor performance in controlled spiked samples, we tested the sensor performance on clinical urine samples obtained from *N* = 30 female human subjects from a post‐menopausal cohort (a mix of both UTI negative and positive patients). For sample testing, 200 μL of unfiltered and unprocessed test urine sample is dropped into the sample loading reservoir. The impedance values for the different samples were quantified using the USENSE system. As seen in Figure 4, UTI threshold classification was done based on the diagnostic thresholds discussed previously to segregate the patients into UTI‐positive and UTI‐negative groups. Using unpaired t‐tests (one‐sided, *α* = 0.05), it was observed that the sensor could differentiate between healthy (UTI negative) and UTI positive cases based on the signal output for (A) IL‐8, (B) PGE2, and (C) LPS levels in the samples.

### 3.7 Specificity

**FIGURE 5:**
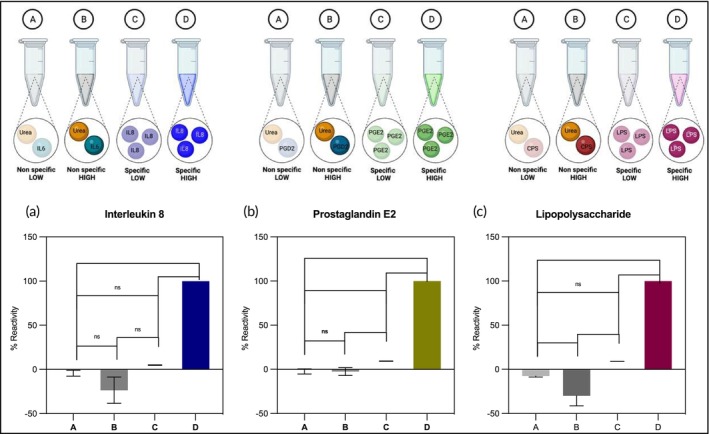
Cross‐reactivity studies for (a) IL8, (b) PGE2, and (3) LPS. A is the cocktail of non‐specific low, B is non‐specific high, C is specific low, and D is specific high molecules. Specific refers to IL8, PGE2, or LPS antigen spiked in artificial urine (pH 6) without urea. Non‐specific low refers to a cocktail solution of non‐specific interferents spiked in artificial urine.

The specificity of the USENSE system was evaluated by studying the response of each of the individual immunogens when challenged with interferent cocktails that contained a structurally similar constituent in urine and urea (the most abundant urine constituent). The PGE2 biosensor was tested with Prostaglandin D2, the IL8 sensor was tested with Interleukin 6, and the LPS sensor was tested with Capsular Polysaccharide for specificity and selectivity. Figure 5 shows the design and the results of the cross‐reactivity experiments. The data is represented with *n* = 9 (*n* = 3 inter‐sensor and *n* = 3 intra‐sensor replicates). A significance test was carried out with α of 0.05. A is the cocktail of non‐specific low, B is nonspecific high, C is specific low, and D is specific high molecules. Specific refers to IL‐8, PGE2, or LPS antigen spiked in artificial urine (pH 6) without urea. Non‐specific low refers to a cocktail solution of non‐specific interferents spiked in artificial urine. The results of the one‐way ANOVA analysis and the Tukey multiple comparisons t‐tests across A–D columns have been discussed in detail in the Data S1 (see Tables S1–S6). There is a significant difference in the sensor response for the same concentration of the specific and non‐specific urine constituents. Thus, from these experiments, it is evident that the signal for PGE2, IL‐8, and LPS does not cross‐react with that for the interferent molecules.

### 3.8 USENSE performance comparison with benchtop

The portable version of the USENSE system was developed using a commercially available multichannel electrochemical potentiostat chip called Emstat MUX 16 (PalmSens, Netherlands). Next, the performance of the portable USENSE sensor was compared with that of the benchtop device (Metrohm Autolab) for multiplexed detection of IL8, PG, E2, and LPS. The signal response for urine samples obtained from *N* = 5 patients was compared (*n* = 3 replicates). Figure S14 shows the results of this experiment. For all the 3 biomarkers, a strong correlation of Pearson's *r* > 0.95 was obtained for the portable USENSE versus the benchtop output. In this way, the translatability of the developed USENSE sensor to a portable point‐of‐care (POC) form factor was confirmed.

### 3.9 Machine learning analysis

The USENSE platform has been designed to give out the diagnosis and the prognosis/relapse prediction for a urine sample at the input. Figure S15 shows the algorithm used in this work. We built two models, one for diagnosis, which was a binary model, and the other was a prognostic model, which gave out a four‐state output based on the truth table discussed in Figure S5. USENSE was used to get the EIS data corresponding to each of the clinical samples. Traditional ELISA was used to establish ground truth and for labeling the samples in the supervised machine learning algorithm (Random Forest model). Figure S16A shows the exploratory analysis of the patient data. Based on that, the state classification was performed. Figure S16B highlights the novelty of USENSE in providing a subclassification of the UTI states in terms of severity and risk of relapse, where the typical urine culture can only provide a Yes or No result.

Specifically, for the diagnosis model, the output states were defined as 0 or 1, corresponding to UTI Negative or UTI Positive, respectively. For the prognosis model, the sensor data acquired from the three biomarkers corresponding to the four Boolean states were labeled as “0,” “1,” “2,” or “3,” corresponding to the output digital state (as discussed in Figure S5). The values of impedance obtained for each target analyte were studied along with the channel in which the target analyte was immobilized.

**FIGURE 6:**
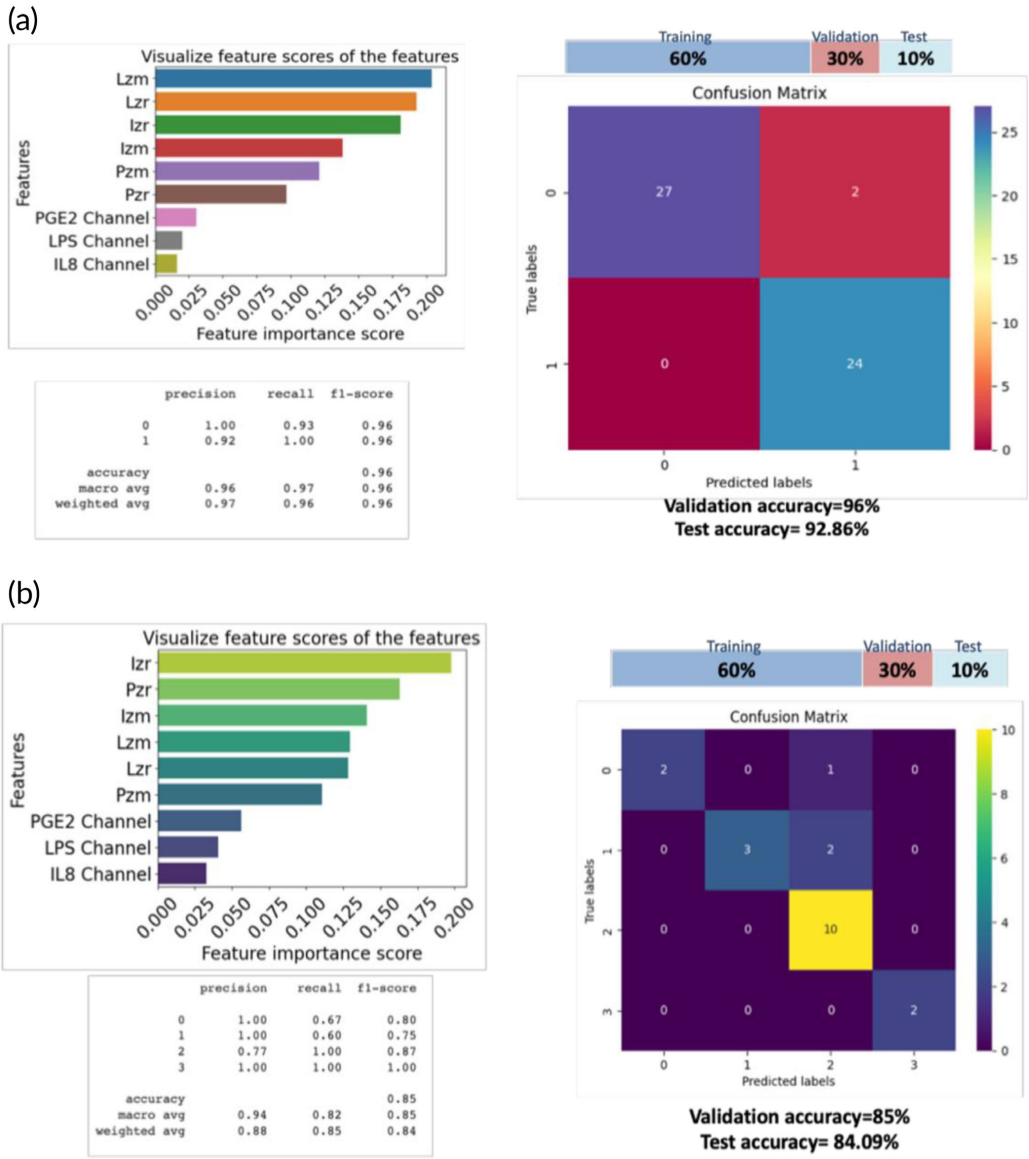
(a) Machine learning results for the UTI Diagnosis model. (b) Machine learning results for the UTI Prognosis model.

Figure 6(a) shows the results of the diagnostic model, while Figure 6(b) shows the results obtained from the prognostic model. For both models, the ratio of training: validation: testing data was 60:30:10. The values of the metrics have also been listed. The diagnostic model showed a high validation accuracy of 96% and a high test accuracy of ~93%. This shows that the model does not suffer from overfitting. For the diagnostic model, the recall value or sensitivity was high for both states: 93% for state 0 and 100% for state 1. This is promising as our model is comparable or possibly better than the current clinical standard urine culture performance, which reportedly has a sensitivity between 50% and 95%. The feature importance scores showed that impedance values of LPS were most important, followed by IL‐8 and PGE2 for UTI diagnosis. The choice of the channel did not affect the model performance, meaning that it did not matter which biomarker assay was immobilized on which channels, thereby validating the “plug‐*n*‐probe” ability of the sensor.

For the prognostic model, a high validation accuracy of 85% and a high test accuracy of ~84% were obtained. A high precision value of >75% was found for all states 0, 1, 2, and 3. In terms of recall, states 2 and 3 were higher than states 0 and 1. The testing accuracy of the developed model has been represented differently in Figure S17, which shows the comparison of actual versus predicted output prognostic states. This figure shows the ability of the model to reliably classify the prognostic states into 0, 1, 2, or 3 using just the top 2 features selected by the model, which are the real impedance parts of Prostaglandin E2 and Interleukin 8. For prognosis, LPS impedance values were not critical. In this way, we developed a first‐of‐a‐kind biosensor platform, which performs a multistate disease classification for UTI prognosis and diagnosis towards home‐based routine screening and self‐triage.

## 4 CONCLUSION

This work demonstrates a novel rapid point‐of‐care testing device for reliable UTI diagnosis by simultaneously evaluating three critical inflammatory biomarkers within 5 minutes using a drop (<0.2 mL) of unprocessed human urine. The device is highly sensitive and specific and is capable of rapidly detecting very low biomarker concentrations. The sensor shows a wide dynamic range, high accuracy, and reliability comparable to established lab standard techniques. Thus, this device can be potentially used as a vital clinical resource that will help doctors make informed clinical decisions in less than 5 minutes (which currently requires at least 3–5 days). We believe that our proposed work is highly novel and can create a paradigm shift in the field of UTI diagnostics and management. By reliably mapping host response due to UTI onset and relapse, we envision that the developed UTI biosensor can transform the current UTI clinical workflow, enabling medical intervention at early stages, which will significantly increase treatment success rates and considerably improve patient outcomes (Figure S23). Future work will include further analysis of other critical parameters for each individual non‐faradaic EIS‐based immunosensor (PGE2, IL‐8, and LPS), such as the stability (inter‐day and intra‐day variability), shelf‐life evaluation (and the need for lyophilization) and reproducibility analysis of USENSE.^46^, ^47^, ^48^, ^49^ Future directions for improving the USENSE platform include expanding the biomarker panel to include additional biomarkers like creatinine and other UTI‐relevant biomarkers to further improve the diagnostic accuracy. The prognostic ability can be further enhanced by including >4 state outcomes. In this work, we have used a simple supervised learning algorithm to demonstrate proof of concept for diagnostic and prognostic performance comparable to the clinical standard. For future versions, more sophisticated supervised or unsupervised algorithms can be explored. The current version of USENSE uses a single sample‐in‐answer‐out model. rUTI is associated with cycles of UTI onset and relapse. To achieve true personalized monitoring and preventive management of rUTI, time series analysis and longitudinal monitoring studies need to be done to validate the sensor performance efficacy and algorithm risk of relapse prediction accuracy over the episodes.

## AUTHOR CONTRIBUTIONS

S. P., A. G., N. J. D., and P. Z. conceived the project framework. S. P. and A. G. designed the experiments, and A. G., V. G., and P. R. performed the experiments. A. G. performed the cleanroom fabrication. A. G. and V. G. optimized the fabrication process. K. J. and A. R. R. performed the batch‐wise sensor cartridge fabrication. A. K. and S. K. developed the graphical user interface and the backend software for getting the readouts and the diagnosis and prognosis outputs. U.B. obtained the data for ELISA validation experiments. A. G., V. G., A. K., U. B., and S. P. wrote the article. N. J. D., P. Z., and U. B. reviewed the manuscript. S. P. and N. J. D. obtained funding.

## FUNDING INFORMATION

This work was funded in part by the Welch Foundation Grant AT‐2030‐20200401 to N. J. D.

## CONFLICT OF INTEREST STATEMENT

S. P. has a significant interest in EnLiSense LLC, a company that may have a commercial interest in the results of this research and technology. The potential individual conflict of interest has been reviewed and managed by the University of Texas at Dallas, and it played no role in the study design, the collection, analysis, and interpretation of data, the writing of the article, or the decision to submit the article for publication.

## Supporting information


**DATA S1:** Supporting Information.

## Data Availability

The authors declare that the data supporting the findings of this study are available within the paper and its Supplementary Information file. Should any raw data files be needed in another format, they are available from the corresponding author upon reasonable request.
