# Supplementary material for: USENSE: A proof‐of‐concept self‐screening tool for home‐based recurrent urinary tract infection management
Source: Bioeng Transl Med. 2025 Jul 10;10(5):e70038. doi: 10.1002/btm2.70038 (PMC12478329; doi:10.1002/btm2.70038)
Supplement: Supplementary file 1 — DATA S1: Supporting Information. [file BTM2-10-e70038-s001.pdf]

USENSE: A proof-of-concept self-screening tool for home-based recurrent Urinary Tract Infection management

Antra Ganguly[1], Ujjaini Basu[2], Varun Gunda[1], Ashwin Krishnan[1], Pranav Ramesh[2], Kush Jivnani[3], Arjun Raghuram[4], Shashank Bhagavatula[2], Sifa Khan[2], Philippe Zimmern[5], Nicole De Nisco[2,5] and Shalini Prasad[1,*]

[1] Department of Bioengineering, University of Texas at Dallas, Richardson, TX, USA.
[2] Department of Biological Sciences, University of Texas at Dallas, Richardson, TX, USA.
[3] Department of Healthcare Management, University of Texas at Dallas, Richardson, TX, USA.
[4] Department of Neuroscience, University of Texas at Dallas, Richardson, TX, USA.
[5] Department of Urology, University of Texas Southwestern Medical Center, Dallas, TX, USA.

Supplementary Information

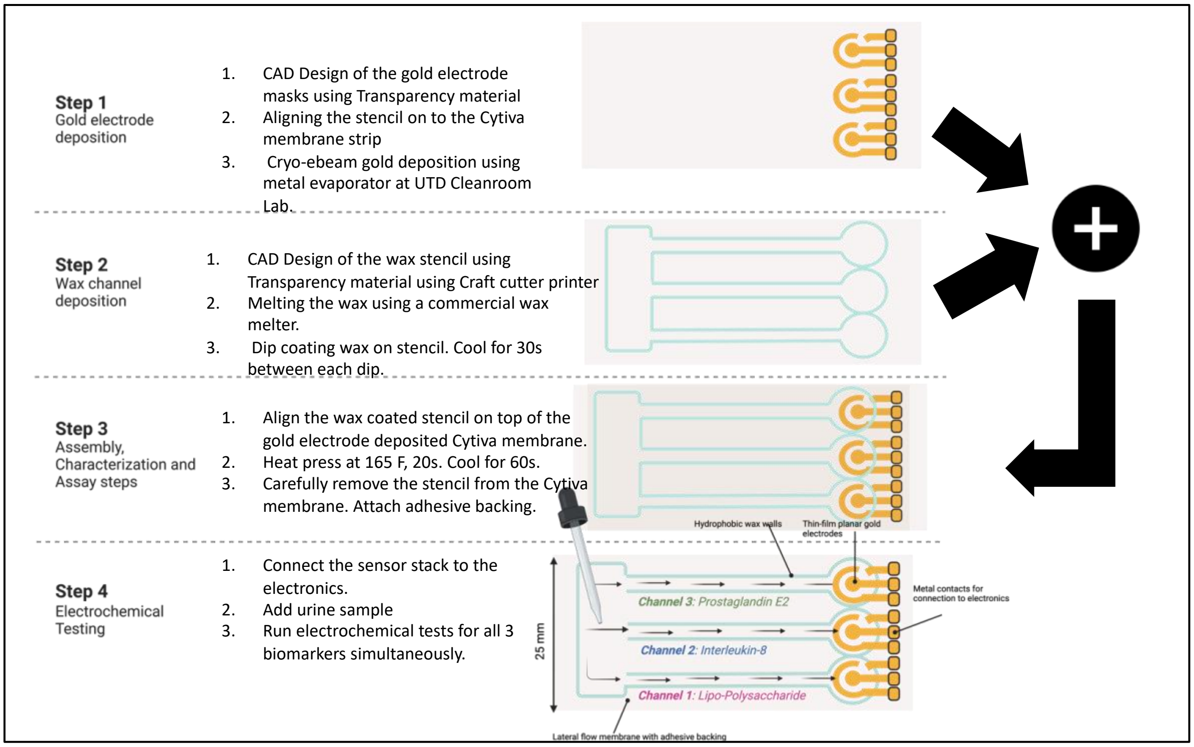

Figure 1: Step by step process flow for USENSE disposable cartridge fabrication.

Fabrication of multiplexed lateral flow sensor

A sensor design was created in AutoCAD with a standard three-electrode system (working, counter, and reference) for each of the three channels. A transparency film sheet was used to cut out the sensor pattern using a Silhouette Cameo 4 cutter printer (Photo paper setting, 6 blade depth, 25 force, 2 passes, 10 speed) to create a stencil for subsequent gold deposition. Cryo e-beam physical vapor deposition using the shadow mask technique (substrate using the patterned transparency sheets as stencils) was done to deposit the gold electrode patterns on the Cytiva Fusion 5. A stencil for the hydrophobic wax barrier (to separate out the individual biomarker channel and corresponding three-electrode system) was prepared in a similar manner (Photo paper setting, 6 blade depth, 25 force, 2 passes, 10 speed in the Silhouette Cameo 4 cutter printer). The cutter printer was loaded with an AutoCAD designed 6 by 7 wax array that delineated the three channels, a primary reservoir, and the area for sensor contacts for each sensor cartridge with the dimensional

constraints of the Fusion 5 membrane (25 mm width). Molten wax was prepared by placing paraffin wax in the wax heater (high setting) for approximately 40 minutes. The printed transparency wax transfer masks were cleaned with IPA and DI water and dipped once in molten wax vertically with the channels facing down so as not to block them. The film sheet masks with accumulated wax were aligned on top of the gold-deposited Fusion 5 membrane strips and taped together. These combined strips were sandwiched between two pieces of aluminum foil in preparation for the heat press. The foiled strips were heat pressed at 175 ºF at maximum pressure for 20 seconds to stamp the wax pattern onto the membrane. The strips were heat pressed and left to cool down at room temperature for 1 minute. The adhesive backing was subsequently applied at the bottom of the stack for structural support to the sensor cartridges.

Optimization and Quality control of the fabrication process

After fabricating the sensors using the protocol discussed in the previous section, baseline characterization was done using ultra-pure deionized water. 0.2 mL of deionized water was dispensed on the sample loading area. The sample flows down the hydrophilic channels and reaches the gold electrodes completing the circuit. The volume of sample required to completely cover the electrodes and complete the electric circuit was found to be 5 μL. This volume was used to calibrate and test the sensors. The impedance modulus (Zmod) at 100 Hz frequency from EIS was recorded for all the 3 channels. Supplementary Figure 2 shows one batch of fabricated sensors that demonstrated a normal distribution across all three channels and were used for the calibration and testing of the sensors. The t-test (two-tailed, unpaired, $\alpha=0.05$) results show that the baseline and the sensor performance are independent of the channel as there is no significant difference ($p>0.05$) between the different impedance components across all 3 channels (Supplementary figure 3).

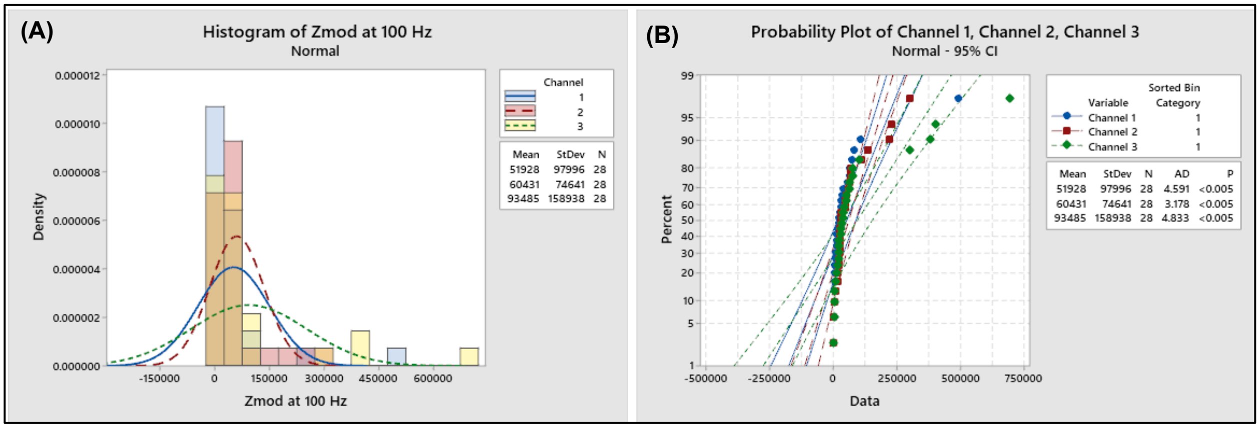

Figure 2: MiniTab software analysis of a batch of fabricated sensor cartridges. (A) Histogram showing the modulus of impedance at 100 Hz and (B) Probability plot of all the 3 channels.

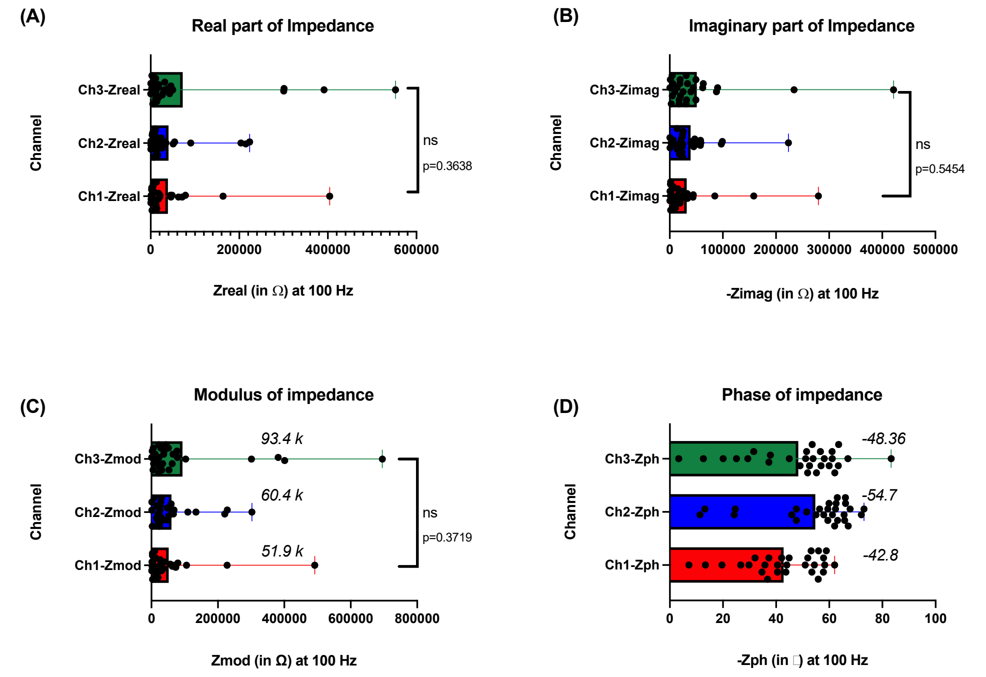

Descriptive Statistics-Summary

	Ch1-Zreal	Ch1-Zimag	Ch1-Zmod	Ch1-Zph	Ch2-Zreal	Ch2-Zimag	Ch2-Zmod	Ch2-Zph	Ch3-Zreal	Ch3-Zimag	Ch3-Zmod	Ch3-Zph
Mean	39141	32096	51928	42.85	40594	39283	60431	54.73	72565	51103	93485	48.36
Std. Deviation	79643	58306	97996	14.37	64177	43938	74641	16.70	137187	85648	158938	17.84
Std. Error of Mean	15051	11019	18520	2.716	12128	8303	14106	3.156	25926	16186	30037	3.371

Figure 3: Distribution of (A) Real, (B) Imaginary, (C) Modulus and (D) Phase of impedance measured at 100 Hz for a batch of fabricated sensors.

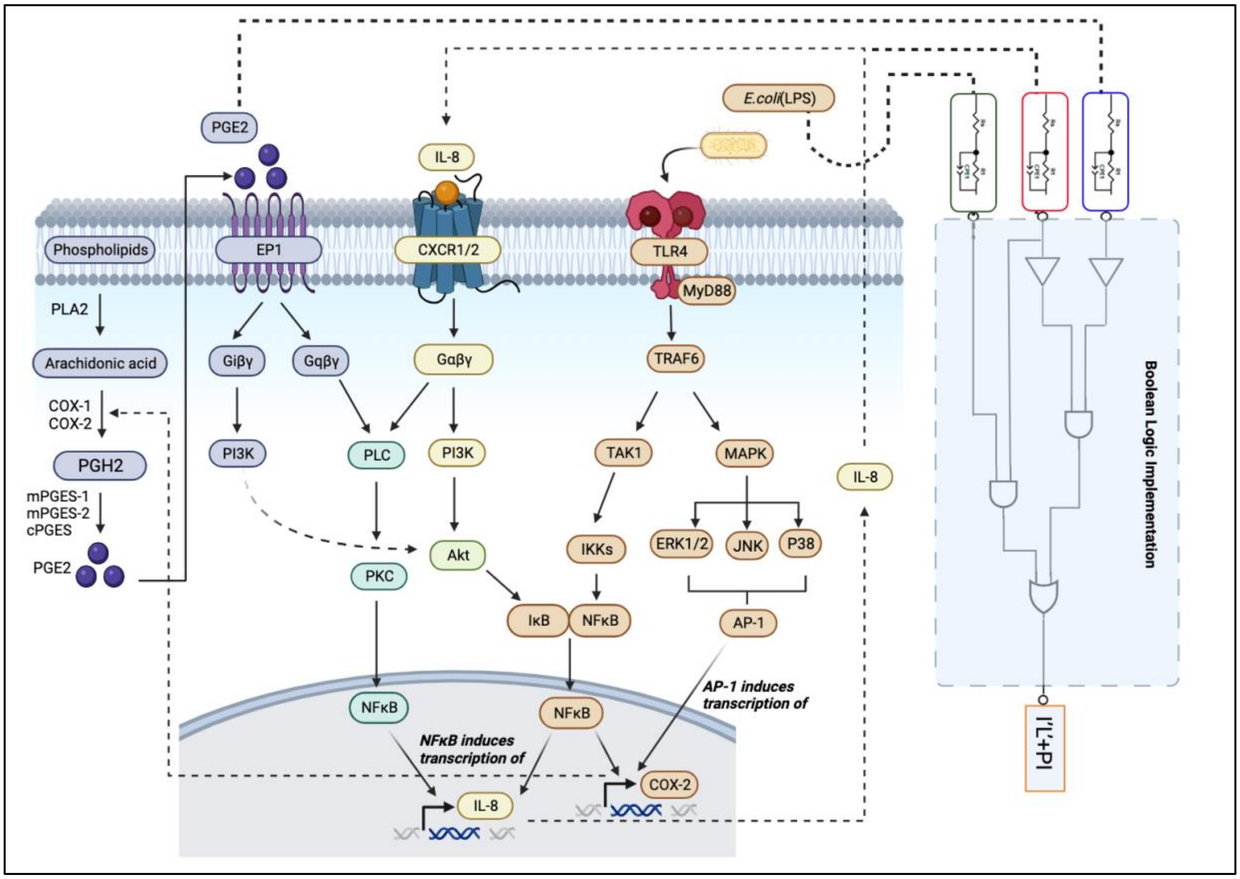

Figure 4: Figure showing the biochemical pathways associated with the target biomarkers viz., IL8, PGE2 and LPS and the Boolean logic governing the UTI diagnosis.

UTI State	LPS*	IL8**	PGE2***	Outcome
0	-	-	-	*UTI Negative*, *Healthy*
1	+	-	-	*UTI Negative*, *Asymptomatic Bacteriuria*
2	+	+	-	*UTI Positive*, *Symptomatic*
3	+	+	+	*UTI Positive*, *Risk of UTI relapse*

*- Bacterial biomarker; **-Diagnostic Inflammatory biomarker **-Prognostic and Diagnostic Inflammatory biomarker;

Figure 5: Truth table guiding the logic for UTI diagnosis and prognosis (risk of relapse prediction).

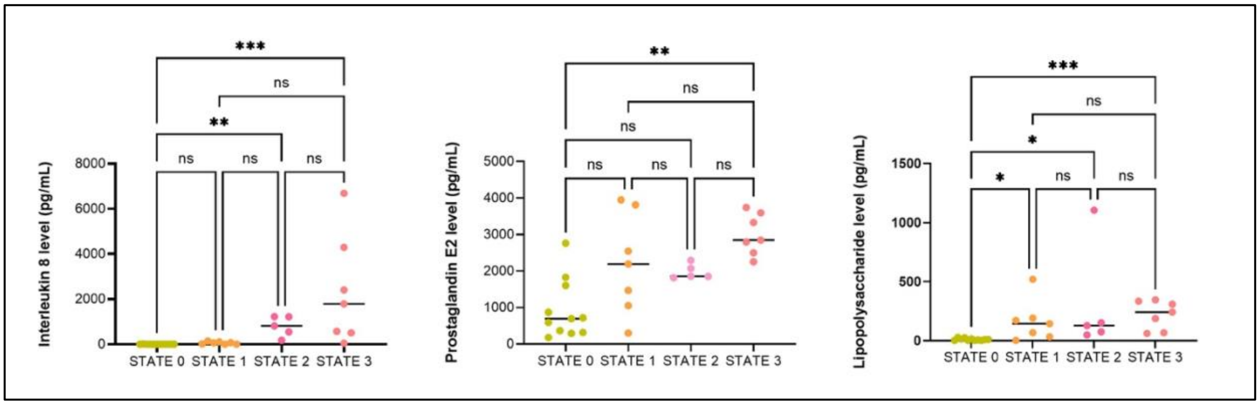

Figure 6: Analysis of the biomarker levels corresponding to the defined states for prognosis and risk of relapse prediction. These values were measured by ELISA.

We used Attenuated Total Reflectance Fourier Transform Infrared spectroscopy (ATR-FTIR) technique to validate the successful formation of the DSP SAM layer and the binding of the DSP to the capture antibodies for each of the immunosensors for the target UTI biomarkers. The results of the ATR-FTIR experiments have been depicted in supplementary figures 7-9. We also performed UV-Vis spectroscopy to validate successful biosensing through antibody-antigen binding. The results have been depicted in supplementary figure 10. The spectrum was obtained from 200-800 nm (data represented for 200-450 nm). Data represented is for Ab-Ag interaction for the highest dose of the target antigen (5000 pg/mL for PGE2, 1000 pg/mL for IL-8, and 50 ng/mL for LPS). For PGE2 and IL-8, the antibody was monoclonal; for LPS, the antibody used was polyclonal.

We also studied the surface charge behavior of the antibody-antigen complex as a function of urine pH using Zeta potential measurement from Dynamic light scattering experiments. Smoluchowski's equation for electrophoretic mobility was utilized(Malvern Instruments, 2011; Sze et al., 2003). The variation in Zeta Potential upon binding of the antigen to the corresponding antibody (highest dose of antigen) was studied (see supplementary figure 11). A stable negative zeta potential was obtained for all the 3 biomarkers (PGE2, LPS and IL-8) indicating that the system is not prone to agglomeration.

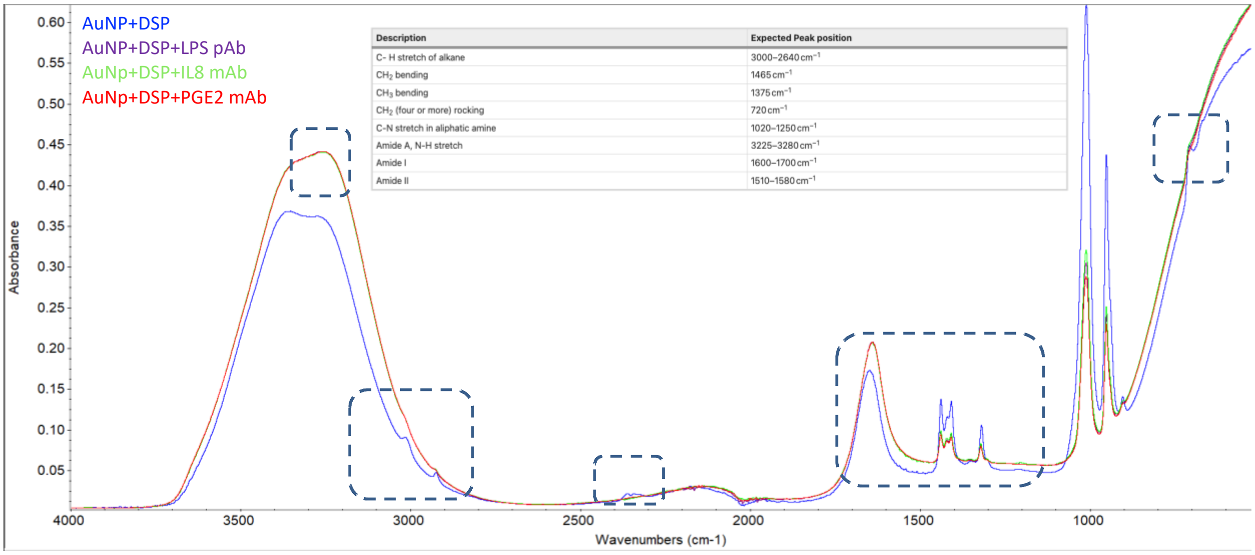

Description	Expected Peak position
C- H stretch of alkane	3000–2640 cm^{-1}
CH_2 bending	1465 cm^{-1}
CH_3 bending	1375 cm^{-1}
CH_2 (four or more) rocking	720 cm^{-1}
C-N stretch in aliphatic amine	1020–1250 cm^{-1}
Amide A, N-H stretch	3225–3280 cm^{-1}
Amide I	1600–1700 cm^{-1}
Amide II	1510–1580 cm^{-1}

Figure 7: ATR-FTIR spectra for all 3 biomarkers and control for 500-4000 cm^{-1}.

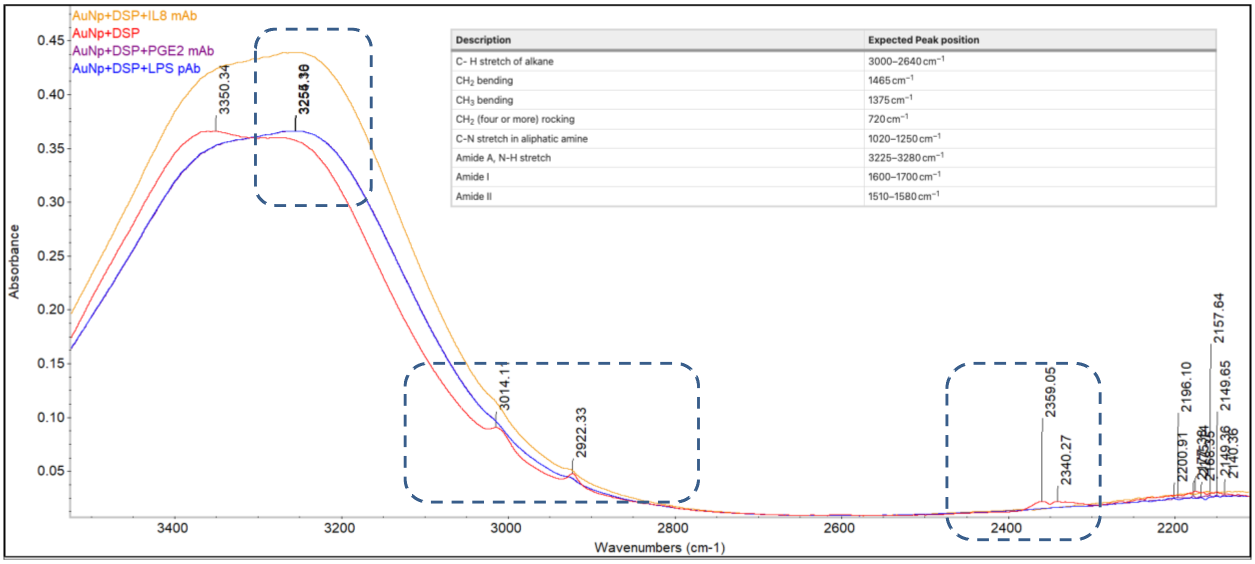

Figure 8: Zoomed in ATR-FTIR spectra for all 3 biomarkers and control for 2000-3500 cm^{-1}.

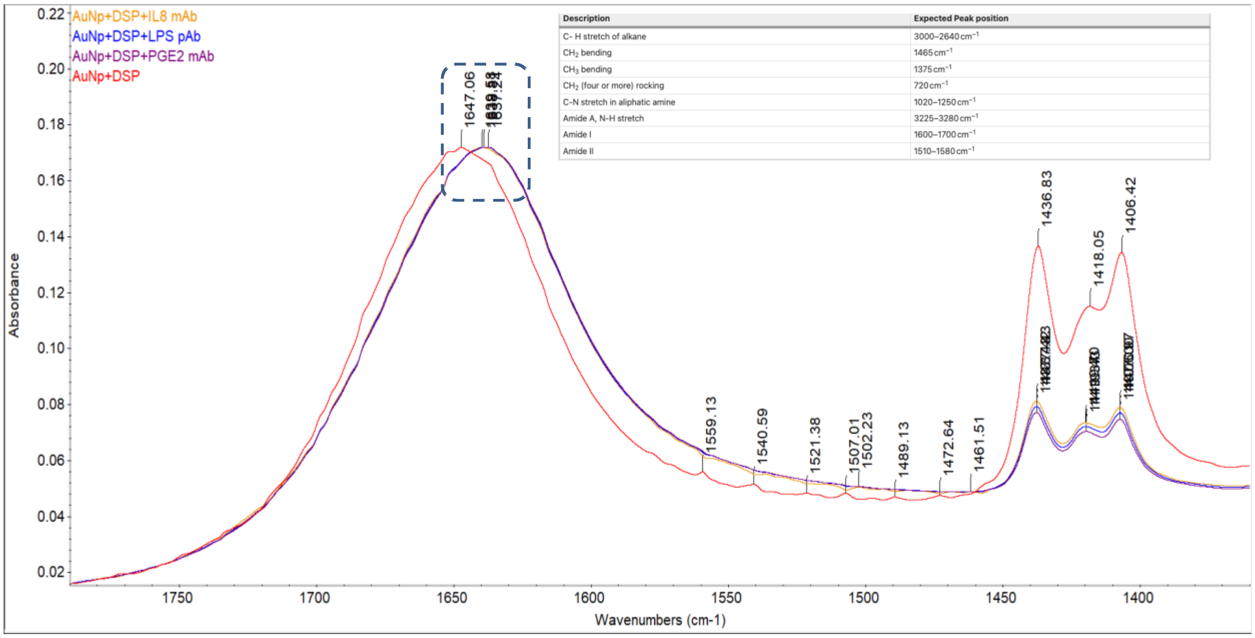

Figure 9: Zoomed in ATR-FTIR spectra for all 3 biomarkers and control for 1300-1800 cm^{-1}.

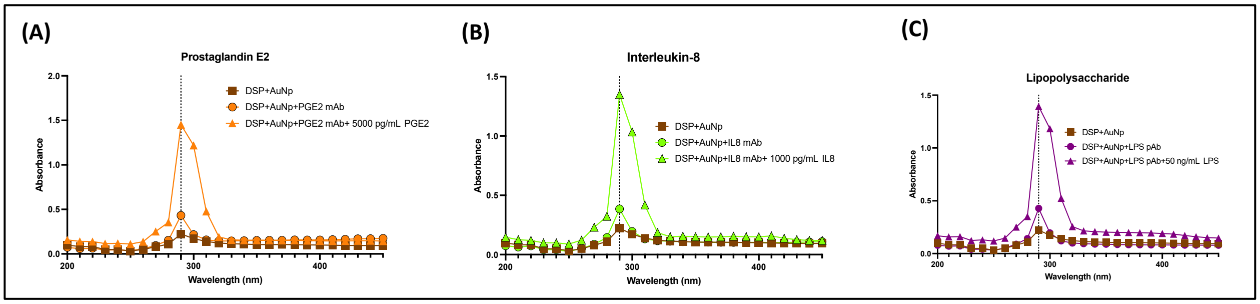

Figure 10: UV-Vis spectra for (A) PGE2, (B) IL8 and (C) LPS assay stack.

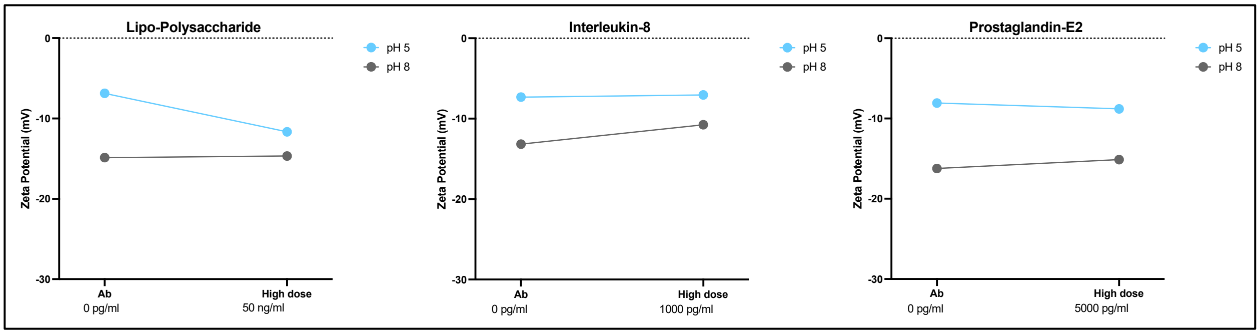

Figure 11: Zeta potential analysis of the surface charge behavior of Ab-Ag binding for all 3 biomarkers for low and high pH.

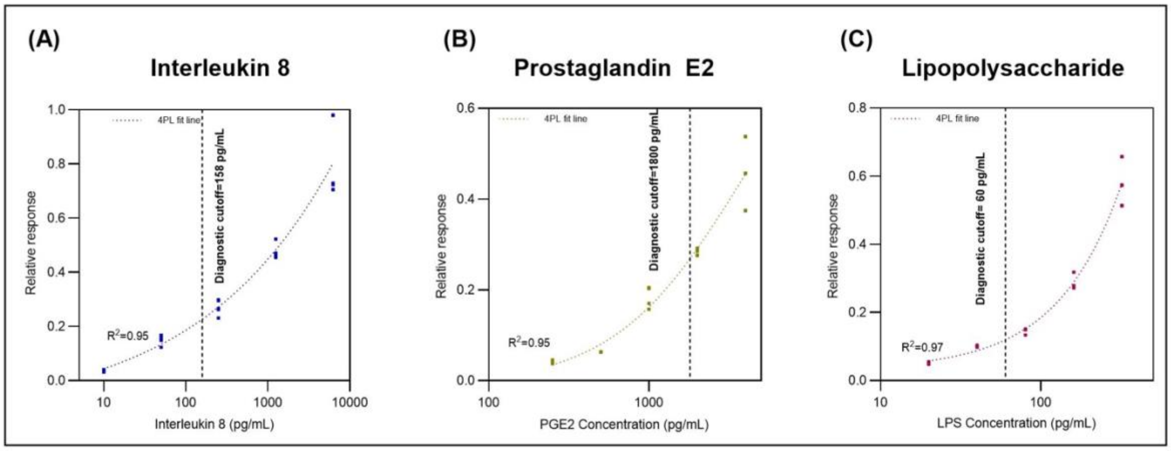

Figure 12: Calibrated dose response curves for (A) IL8, (B) PGE2, and (C) LPS.

The relative response is measured as the percent change values for different doses in the calibration dose response for spiked pooled human urine studies calculated relative to the zero-dose/blank value i.e. the EIS response to zero dose/ baseline dose i.e., 0 pg/mL of biomarker in the un-spiked/raw pooled human urine sample. The percent changes are calculated based on the following formula:

Percentage change in modulus of impedance at 100 Hz =

$$\frac{(\text{Modulus of impedance measured for Blank} - \text{Modulus of impedance measured for spiked sample}}{\text{Modulus of impedance measured for Blank}} * 100$$

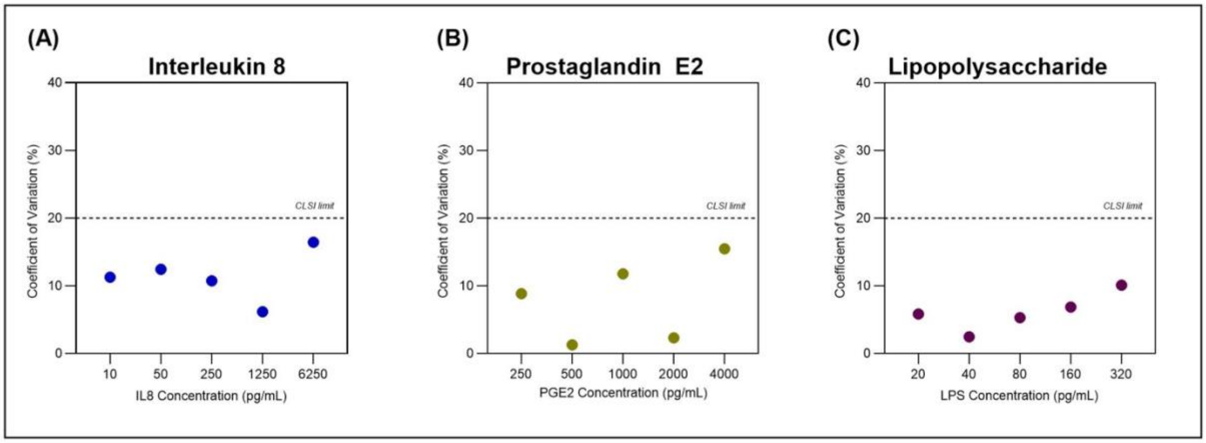

Figure 13: CV% analysis for (A) IL8, (B) PGE2 and (C) LPS.

Crossreactivity study and Specificity analysis

Cross-reactivity studies were done for (A) IL8, (B) PGE2, and (3) LPS. A is the cocktail of non-specific low, B is nonspecific high, C is specific low, and D is specific high molecules. Specific refers to IL8, PGE2 or LPS antigen spiked in artificial urine (pH 6) without urea. Non-specific low refers to a cocktail solution of non-specific interferents spiked in artificial urine. Significance test was carried out with α of 0.05. A is the cocktail of non-specific low, B is nonspecific high, C is specific low, and D is specific high molecules. Specific refers to IL-8, PGE2, or LPS antigen spiked in artificial urine (pH 6) without urea. Non-specific low refers to a cocktail solution of non-specific interferents spiked in artificial urine. The results of 1-way ANOVA analysis and the Tukey multiple comparisons t-tests across A-D columns have been tabulated in Tables 1-6 below. There is a significant difference in the sensor response for the same concentration of the specific and non-specific urine constituents. Thus, from these experiments, it is evident that the signal for PGE2, IL-8, and LPS does not cross-react with that for the interferent molecules.

Table 1: One way ANOVA analysis for IL-8 sensor

F	52.05
P value	<0.0001
P value summary	****
Significant diff. among means (P < 0.05)?	Yes
R squared	0.8299

Table 2: Tukey multiple comparison t-test analysis for IL-8 sensor

Tukey's multiple comparisons test	Mean Diff.	95.00% CI of diff.	Below threshold?	Summary	Adjusted P Value	
A vs. B	19.25	-10.03 to 48.53	No	ns	0.3005	A-B
A vs. C	-9.211	-38.49 to 20.07	No	ns	0.829	A-C
A vs. D	-104.3	-133.6 to -75.05	Yes	****	<0.0001	A-D
B vs. C	-28.46	-57.74 to 0.8141	No	ns	0.0591	B-C
B vs. D	-123.6	-152.9 to -94.30	Yes	****	<0.0001	B-D
C vs. D	-95.12	-124.4 to -65.84	Yes	****	<0.0001	C-D

Table 3: One way ANOVA analysis for PGE2 sensor

F	344.8
P value	<0.0001
P value summary	****
Significant diff. among means (P < 0.05)?	Yes
R squared	0.97

Table 4: Tukey multiple comparison t-test analysis for PGE2 sensor

Tukey's multiple comparisons test	Mean Diff.	95.00% CI of diff.	Below threshold?	Summary	Adjusted P Value	
A vs. B	0.2072	-10.02 to 10.44	No	ns	>0.9999	A-B
A vs. C	-11.61	-21.84 to -1.386	Yes	*	0.0211	A-C
A vs. D	-102.3	-112.5 to -92.09	Yes	****	<0.0001	A-D
B vs. C	-11.82	-22.05 to -1.593	Yes	*	0.0185	B-C
B vs. D	-102.5	-112.8 to -92.30	Yes	****	<0.0001	B-D
C vs. D	-90.7	-100.9 to -80.47	Yes	****	<0.0001	C-D

Table 5: One way ANOVA analysis for LPS sensor

F	97.79
P value	<0.0001
P value summary	****
Significant diff. among means (P < 0.05)?	Yes
R squared	0.9017

Table 6: Tukey multiple comparison t-test analysis for LPS sensor

Tukey's multiple comparisons test	Mean Diff.	95.00% CI of diff.	Below threshold?	Summary	Adjusted P Value	
A vs. B	22.37	0.2649 to 44.48	Yes	*	0.0465	A-B
A vs. C	-16.41	-38.51 to 5.701	No	ns	0.2053	A-C
A vs. D	-107.6	-129.7 to -85.49	Yes	****	<0.0001	A-D
B vs. C	-38.78	-60.88 to -16.67	Yes	***	0.0002	B-C
B vs. D	-130	-152.1 to -107.9	Yes	****	<0.0001	B-D
C vs. D	-91.19	-113.3 to -69.09	Yes	****	<0.0001	C-D

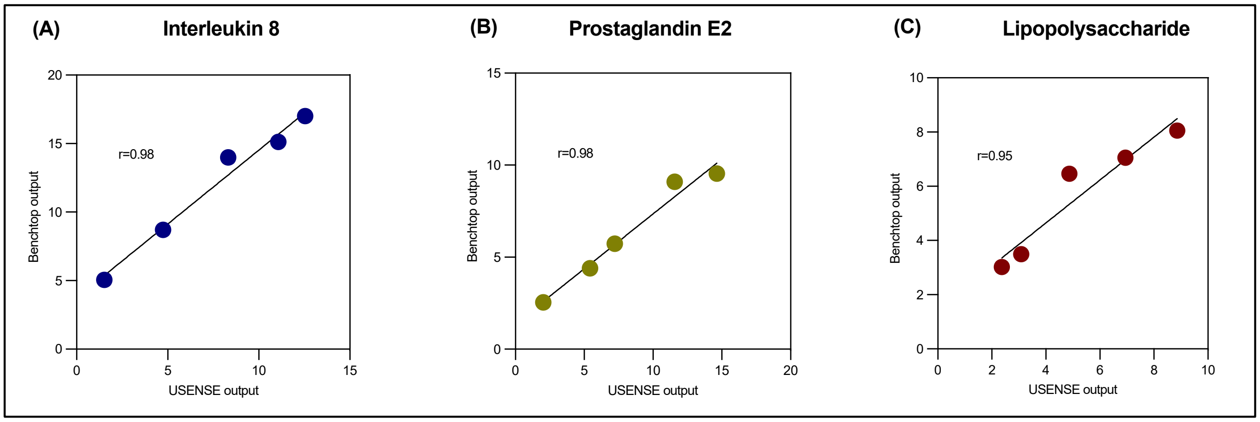

Figure 14: Correlation analysis of sensor output for benchtop versus portable USENSE device for (A) IL8, (B) PGE2 and (C) LPS.

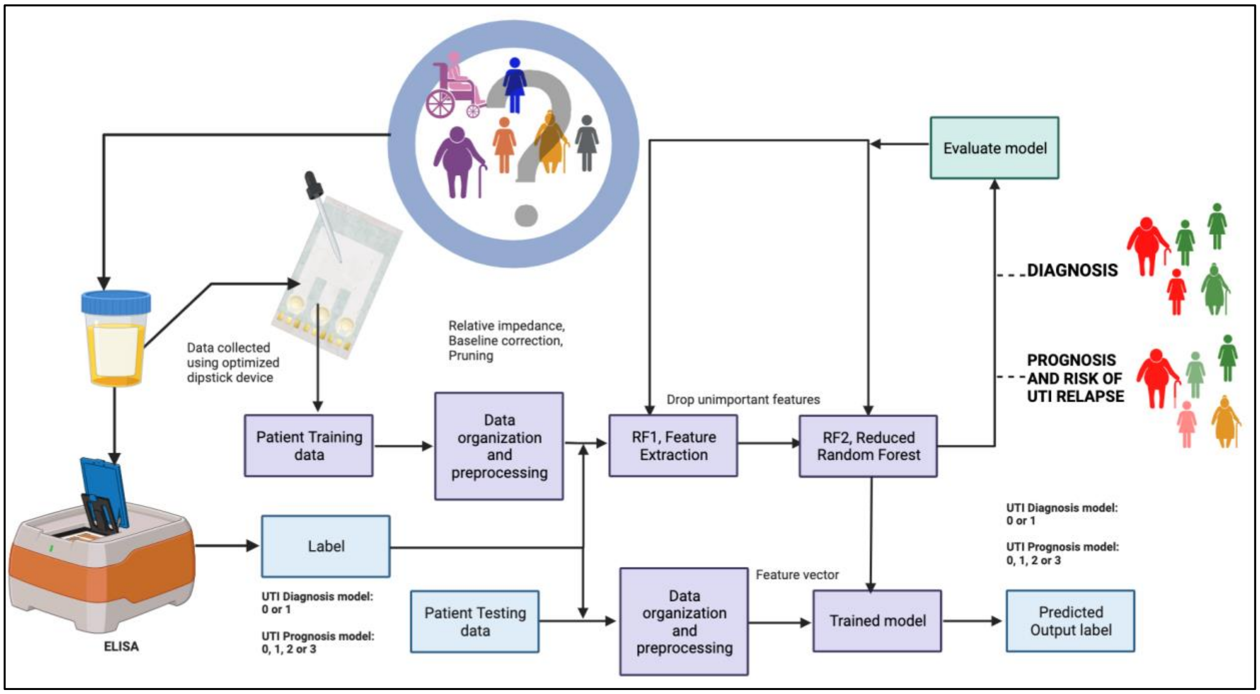

Figure 15: Flowchart describing the design of machine learning analyses.

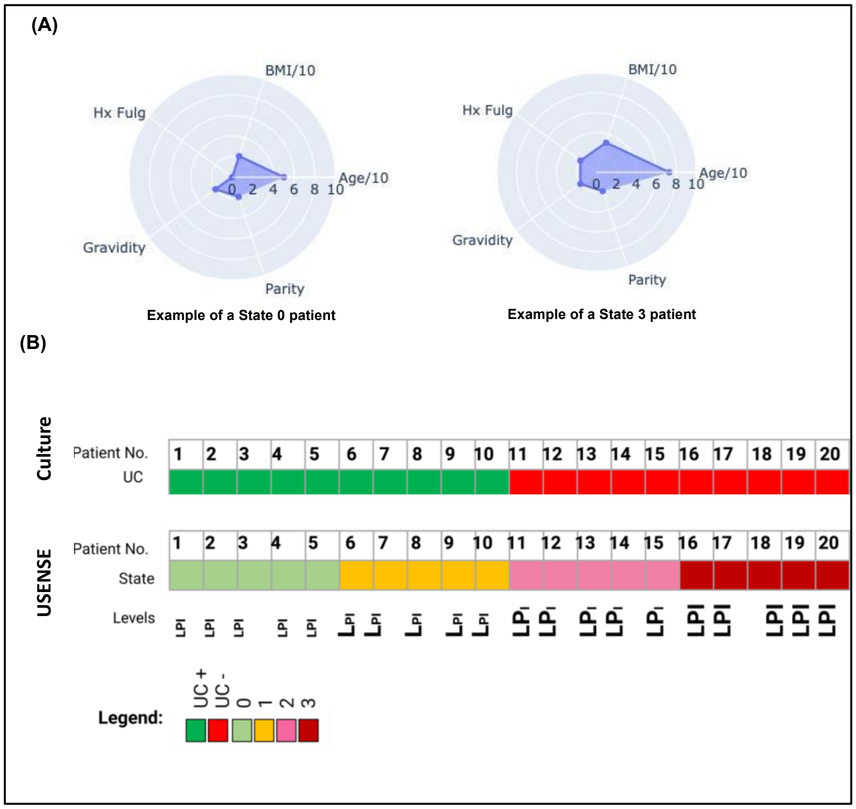

Figure 16: (A) Exploratory analysis of patient metadata and (B) UTI state subclassification capability of USENSE.

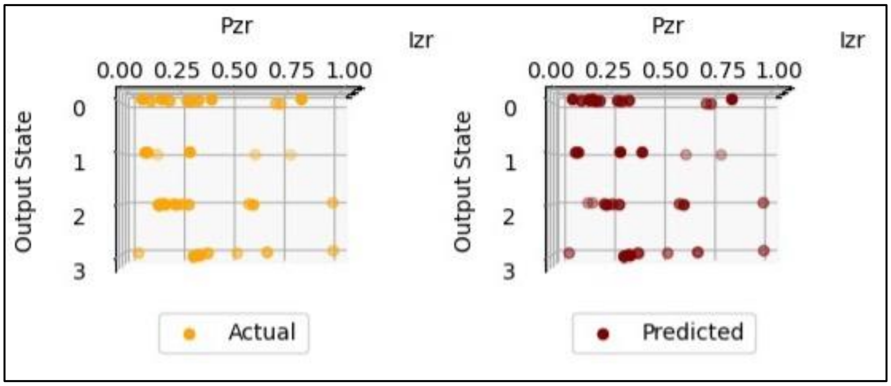

Figure 17: Comparison of actual versus predicted output prognostic states.

Electronic system design and integration

GUI design and EIS analysis through an EmStat Pico MUX16

This software implements the PalmSens WinForms SDK through the Unity game engine to create an environment in which multichannel EIS (Electrochemical Impedance Spectroscopy) results can be read and analyzed through a single application (supplementary figure 16). The application can be run by connecting a PalmSens EmStat MUX 16 to any device (with Windows OS) and locally saves all pertaining clinical information for easy access.

System Integration and Compatibility

The USENSE cartridge features a three-electrode design – a working electrode (WE), reference electrode (RE), and counter electrode (CE). The WE recorded the electrochemical characteristics of the analyte, focusing on potential and current variations. The simultaneous operation across multiple channels facilitates parallel measurements, which are relayed through the multiplexer. Communication with the potentiostat and multiplexer is done using MethodScript. This script is incorporated into the GUI by attaching its code as a separate application. The instructions for use for the USENSE platform has been depicted in supplementary figure 20 below.

PalmSens WinForms SDK

The PalmSens WinForms SDK is used to communicate with the EmStat using C#. Methodscript is passed as a string and implemented using C# through the MethodScript DLL, which starts EIS with its specifications. EIS results are then read and displayed on a plot in a WinForms window using methods derived from the SDK.

Graphical User Interface (GUI)

This GUI was developed utilizing the Unity game engine (supplementary figures 17, 18 and 19). Objects are attached to scripts, which are organized in a hierarchical fashion. Below are some of the objects and their functions in the GUI.

1. New Test: Runs a new EIS measurement, and saves the average value of Zmod
2. History: Opens an excel file of each patient that has been archived
3. Recalibrate: Opens an excel file of the calibrated parameters used to convert the measured impedances into biomarker concentration levels.
4. Information: Opens a panel giving user instructions on how to use the application.
5. Archive: Archives the patient data file, which is accessible through the history tab. The patient ID, Notes, LPS, PGE2, and IL8 measurements are all saved locally
6. Clear History: Scrubs the patient information file clean

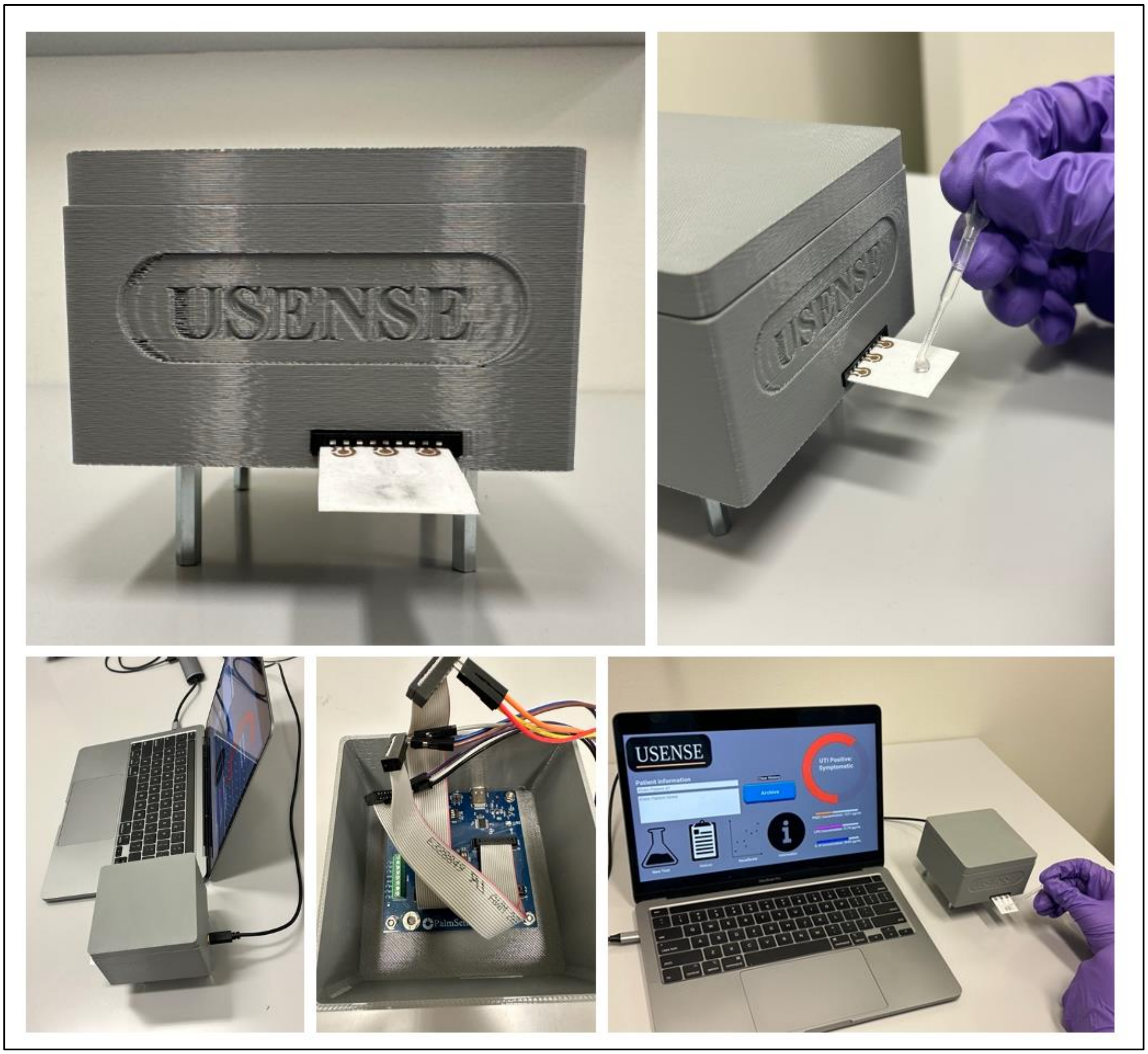

Figure 18: End to end USENSE platform. Figure shows the 3D printed reader encapsulating the Emstat MUX 16 multichannel potentiostat. The cartridge is slotted in the reader as shown and the urine sample is dropped onto the disposable cartridge. The reader is connected to the computer/ laptop via USB communication. The GUI has been programmed to output the UTI diagnosis and prognosis results.

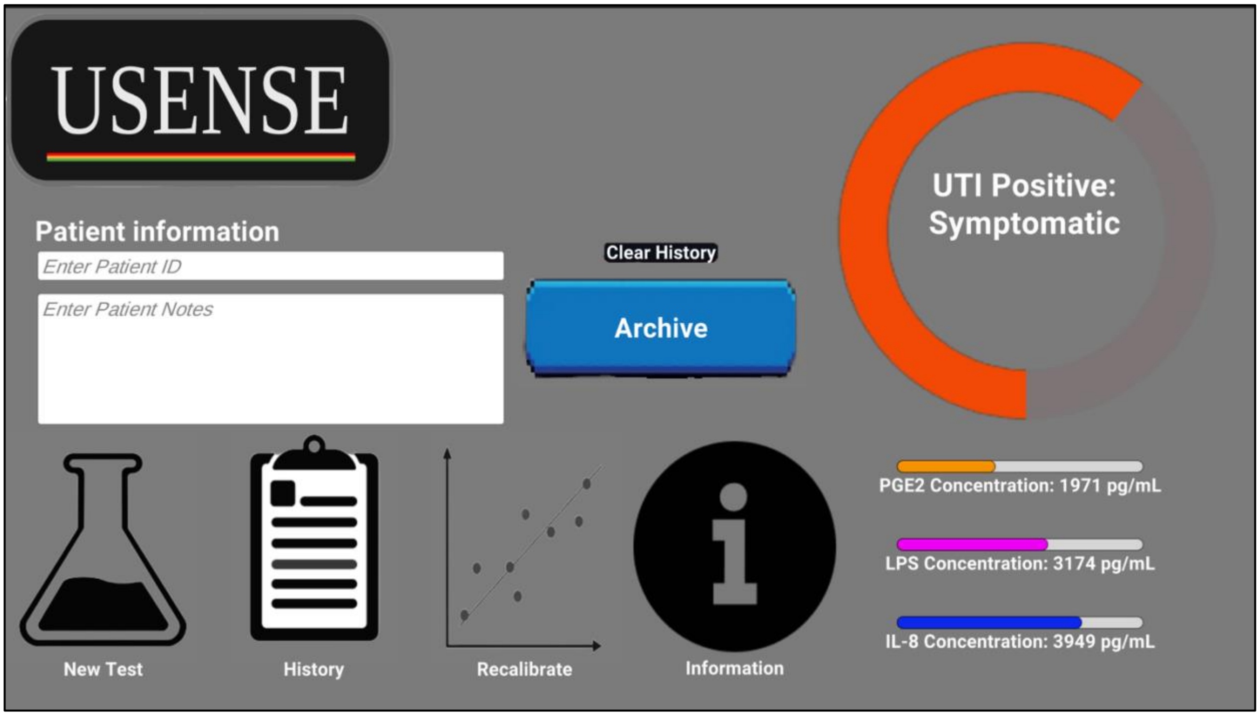

Figure 19: Graphical User Interface for the USENSE system created using Unity program.

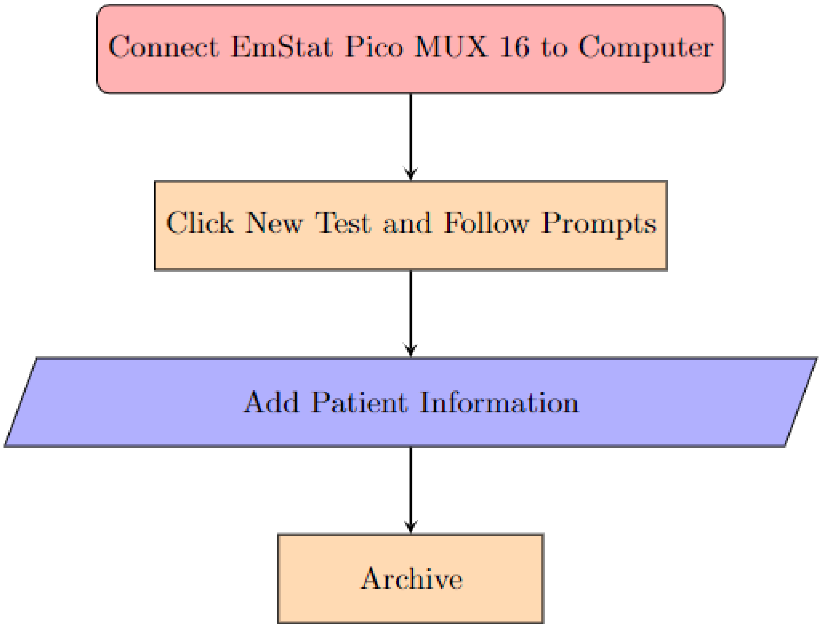

Figure 20: Workflow for general use of Application

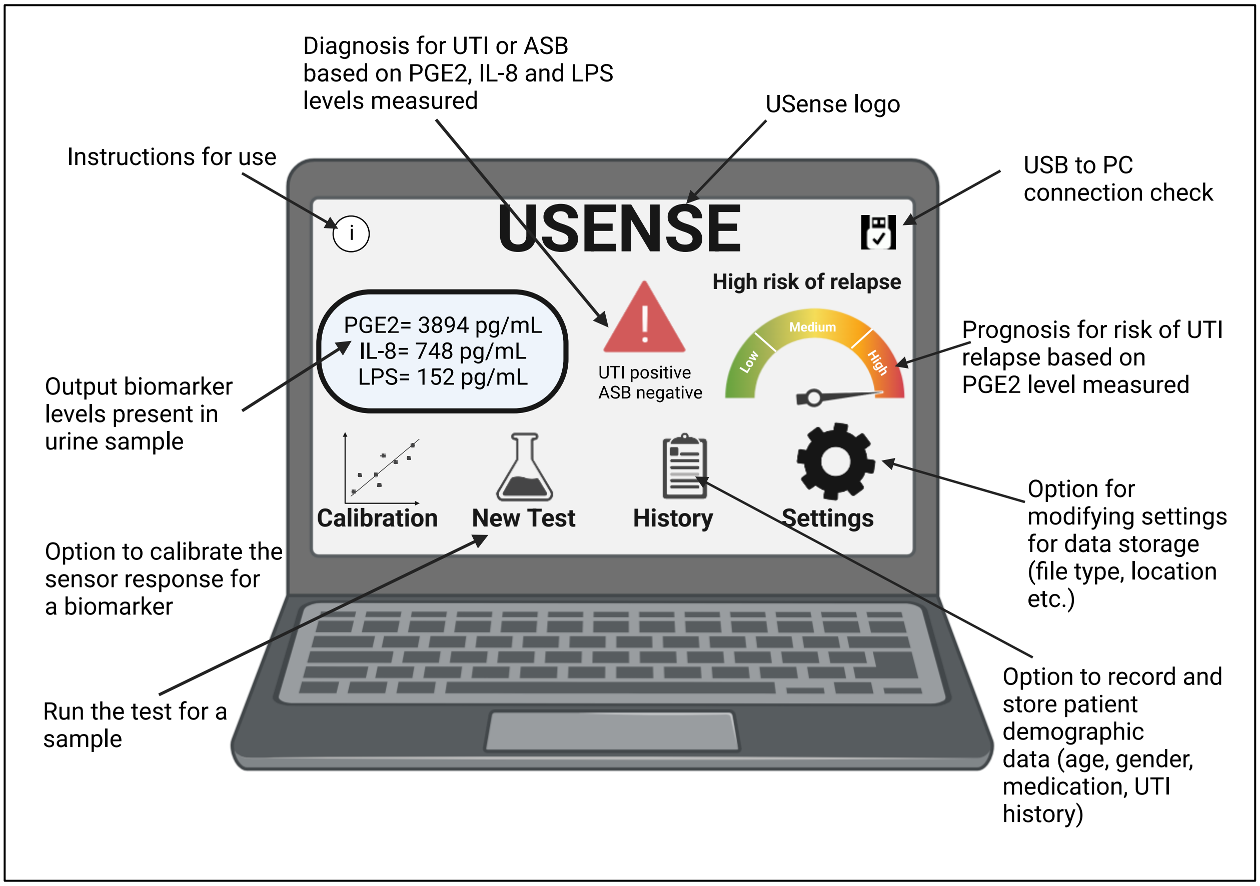

Figure 21: Schematic showing the USENSE Graphical User Interface design.

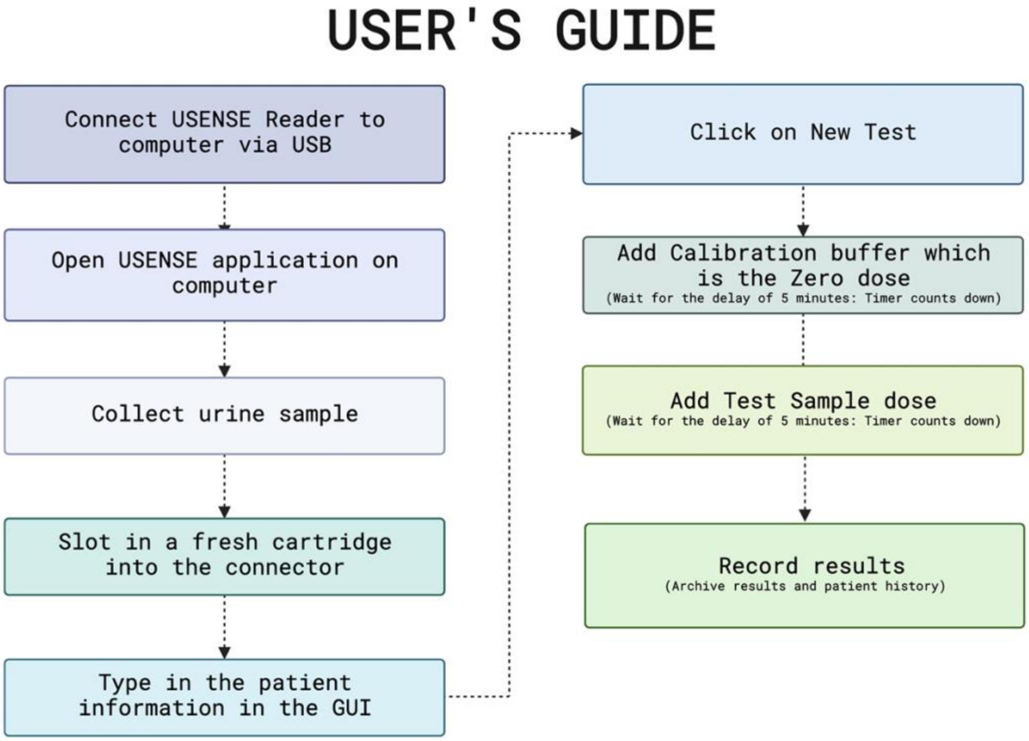

Figure 22: Instructions for use of USENSE platform.

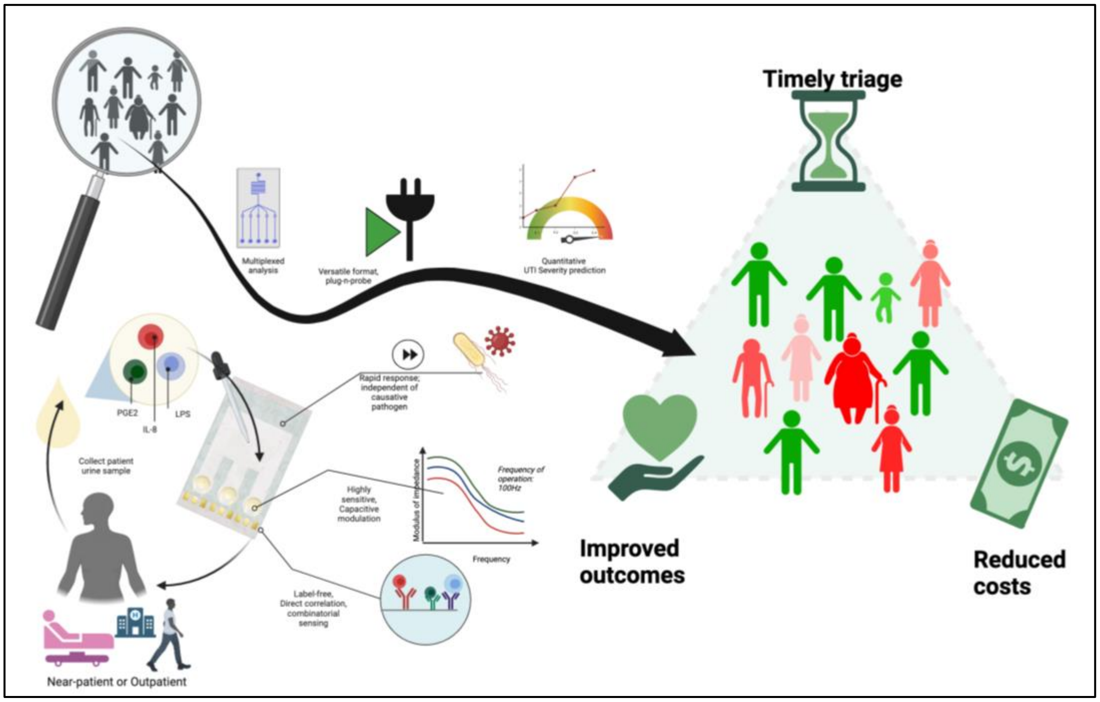

Figure 23: Schematic showing the attributes of the developed USENSE platform and its implications.
